# QTL mapping and candidate gene analysis of ferrous iron and zinc toxicity tolerance at seedling stage in rice by genome-wide association study

**DOI:** 10.1186/s12864-017-4221-5

**Published:** 2017-10-27

**Authors:** Jian Zhang, Kai Chen, Yunlong Pang, Shahzad Amir Naveed, Xiuqin Zhao, Xiaoqian Wang, Yun Wang, Michael Dingkuhn, Julie Pasuquin, Zhikang Li, Jianlong Xu

**Affiliations:** 1 Institute of Crop Sciences/National Key Facility for Crop Gene Resources and Genetic Improvement, Chinese Academy of Agricultural Sciences, Beijing, 100081 China; 2 Agricultural Genomics Institute, Chinese Academy of Agricultural Sciences, Shenzhen, 518120 China; 3 Cirad, Umr AGAP (Dept. BIOS) and Upr AIDA (Dept. ES), F-34398 Montpellier, France; 4 IRRI, CESD Division, DAPO Box 7777, Metro Manila, Philippines; 5 Shenzhen Institute of Breeding and Innovation, Chinese Academy of Agricultural Sciences, Shenzhen, 518120 China

**Keywords:** Rice, Fe toxicity, Zn toxicity, GWAS, QTL

## Abstract

**Background:**

Ferrous iron (Fe) and zinc (Zn) at high concentration in the soil cause heavy metal toxicity and greatly affect rice yield and quality. To improve rice production, understanding the genetic and molecular resistance mechanisms to excess Fe and Zn in rice is essential. Genome-wide association study (GWAS) is an effective way to identify loci and favorable alleles governing Fe and Zn toxicty as well as dissect the genetic relationship between them in a genetically diverse population.

**Results:**

A total of 29 and 31 putative QTL affecting shoot height (SH), root length (RL), shoot fresh weight (SFW), shoot dry weight (SDW), root dry weight (RDW), shoot water content (SWC) and shoot ion concentrations (SFe or SZn) were identified at seedling stage in Fe and Zn experiments, respectively. Five toxicity tolerance QTL (*qSdw3a*, *qSdw3b*, *qSdw12* and *qSFe5* / *qSZn5*) were detected in the same genomic regions under the two stress conditions and 22 candidate genes for 10 important QTL regions were also determined by haplotype analyses.

**Conclusion:**

Rice plants share partial genetic overlaps of Fe and Zn toxicity tolerance at seedling stage. Candidate genes putatively affecting Fe and Zn toxicity tolerance identified in this study provide valuable information for future functional characterization and improvement of rice tolerance to Fe and Zn toxicity by marker-assisted selection or designed QTL pyramiding.

**Electronic supplementary material:**

The online version of this article (10.1186/s12864-017-4221-5) contains supplementary material, which is available to authorized users.

## Background

Ferrous iron (Fe) and zinc (Zn) are essential trace elements for rice, as they are involved in numerous physiological and biochemical processes. In trace amounts, these two elements serve as pivotal cofactors for many enzymes and key structural motifs for transcriptional regulatory proteins. However, due to natural and industrial processes, Fe and Zn occurring in high quantities cause heavy metal toxicity that greatly affect rice growth and quality [1]. Fe toxicity is a serious constraint to the production of lowland rice grown in acid flooded soils [2]. Reported yield losses in fields usually range between 15 and 30%, but can also cause complete crop failure [3]. Zn toxicity can occur in acid soil and is extremely harmed to the growth of rice plants [4]. Moreover, high concentration of Fe and Zn in the soil may lead nutrient imbalance by limiting the absorption of other nutrients. Many researchers have studied the toxic effect of Fe [2, 5] and Zn [6, 7] on rice. Fe and Zn toxicities have similar phenotypic effects and both can result in oxidative cell damage accompanied by the induction of antioxidative defense mechanisms. The typical visual symptom caused by Fe toxicity is small brown-purple spots, appearing from the tips towards the base of the older leaves, commonly known as “bronzing” [8]. Zn toxicity symptoms are first characterized by leaf discoloration and bronzing of older leaves that then spread to the whole plant [9]. Plants have developed several resistance mechanisms such as restricted uptake, extrusion, chelation, trafficking, and storage to maintain essential trace element homeostasis and alleviate heavy metal toxicity [10].

Breeding resistant varieties is an economically sustainable solution to improve rice production under Fe and Zn stress conditions. The genetic variation for Fe and Zn toxicity tolerance in rice is controlled by several genes and its expression largely depends on the environment [8, 11]. Several genes responsible for the uptake, transport and accumulation of Fe and Zn have been identified in rice, which belong to five known protein families (*OsNRAMPs*, *OsFROs*, *OsZIPs*, *OsFERs* and *OsYSLs*) [12]. Many QTL for Fe or Zn toxicity tolerance in rice have been identified and mapped using DNA molecular markers in the populations derived from two parents [8, 9, 13–17]. These QTL are associated with some easily measurable traits like leaf bronzing index, shoot height, root length, shoot and root dry weight, tiller number under toxicity stress, because physiological process traits are difficult to measure and map at the whole population level [18]. Considering that biparental populations in linkage mapping only evaluate two alleles and provide limited insight into the analysis of complex traits unless the population is very large [19], association mapping is becoming an alternative method for dissecting complex traits controlled by multiple QTL and can evaluate a greater number of alleles in a broader population [20]. Due to strong interaction effects of genotype and environment in the field, studies of Fe and Zn tolerance, selection or testing of tolerance in hydroponics solution is a quicker and more efficient way to determine tolerant and sensitive lines in rice while controlling other environmental effects. To our best knowledge, few reports have conducted GWAS for dissecting complex traits related to Fe and Zn toxicity tolerance in rice so far [21].

Understanding of genetic and molecular mechanisms underlying Fe and Zn tolerance in rice is essential to accelerate the development of Fe and Zn tolerant varieties. The objectives of this study were the following: 1) to screen for Fe and Zn toxicity tolerance of a world-wide *Oryza sativa indica* subspecies; and 2) to identify QTL related to Fe and Zn toxicity tolerance.

## Methods

### Plant materials

A total of 222 *indica* rice accessions were introduced from IRRI, which were collected from 31 countries in Asia, Africa and Latin America. It was a subset taken from core collection established at the International Rice Research Institute (IRRI) and represents a well selected panel of phenotypically diverse lines for many agronomic traits. As previously indicated [22], two subgroups of the 222 associations were found by PCA 3 dimension plot (Additional file 1). Given the strong population differentiation, 11 accessions were removed and the remaining 211 accessions were used for the following analysis in this study.

### Phenotypic evaluation

The experiments were carried out in greenhouse of Institute of Crop Sciences, Chinese Academy of Agricultural Sciences in Beijing. Zn toxicity experiment was conducted from May to June and Fe toxicity experiment was from August to September in 2016. Randomized complete block design (RCBD) consisting of 211 accessions (10 plants per accession) with 2 replicates were applied for both control and stress conditions. For zinc toxicity experiment, the temperature was around 28/20 °C (day/night) and the relative humidity was ~60%. For ferrous iron toxicity experiment, the temperature was around 32/25 °C (day/night) and the relative humidity was ~75%. The growth condition in greenhouse was regulated by using shade net and mechanical ventilation along with the cooling pad.

The seeds were surface sterilized with 5% sodium hypochlorite solution for 20 min and rinsed well with distilled water. Then seeds were soaked in distilled water in the dark at 30 °C for 48 h. Finally, 10 uniformly germinated seeds of each accession were directly sown in holes of perforated styrofoam sheets (10 lines × 13 rows) with a nylon net bottom in a plastic container. The styrofoam sheets were allowed to float on water up to five days and then transferred to Yoshida solution [23] for five days. At three-leaf stage, the Fe in the form of FeSO_4_ • 7H_2_O at the concentration of 300 mg L^−1^ (5.36 mM) (2.0 mg L^−1^ for control) or the Zn in the form of ZnSO_4_ • 7H_2_O at the concentration of 200 mg L^−1^(3.06 mM) (0.01 mg L^−1^ for control) were applied. The pH of the solution was adjusted to 5.0 at alternative day by 1 M NaOH / HCl. The solution was renewed every five days. After 21 days of treatment, the shoot height (SH), maximum root length (RL), and shoot fresh weight (SFW) of each plant was measured. Then the samples were kept in oven for 72 h under 50 °C. Finally shoot dry weight (SDW) and root dry weight (RDW) were recorded, and shoot water content (SWC) was determined by (SFW– SDW) / SFW × 100. For each trait, its stress tolerance index was calculated by trait ratio of stress to control condition. The average value of two replicates was taken for all traits.

### Measurement of plant Fe and Zn concentrations

The concentration of Fe or Zn in shoot (SFe or SZn) samples under stress conditions were determined by atomic absorption spectrometry (AAS, Series2, Thermo Electron Corporation) with wet digestion method (GB/T 14609–2008). About 1 g dried shoot samples from each lines was digested with 5 ml mix acid (HNO_3_:HClO_4_ = 4:1, *V*/V) using a graphite liquation furnace. The heating process was as follows: 80 °C for 15 min, 120 °C for 20 min, 150 °C for 30 min and 180 °C for 60 min. Finally, the colorless or slightly yellow transparent liquid was diluted in 100 ml volumetric flask with distilled water. For Fe and Zn determinations, calibration standard solutions were prepared by diluting 1000 μg ml^−1^ standard solution (NCS, China). Two replicates were performed per sample and the average value was taken.

### Genotyping

The genotyping data used in this study was from a high-density rice array (HDRA) composed of 700,000 single-nucleotide polymorphisms (SNPs). The HDRA was developed as an Affymetrix Custom GeneChip Array from a SNP discovery dataset consisting of ~16 millions SNPs (generated by re-sequencing 128 rice samples at ~7X genome coverage). Methods for the development of the HDRA, including SNP discovery and selection, probe design, genotype calling and quality control were described by McCouch [24]. The SNP with minor allele frequency (MAF) less than 0.05 were removed, and finally 395,553 SNP markers were selected for GWAS.

### Data analysis and QTL mapping

The Spearman’s rank correlation coefficients between each trait pair were calculated by the SAS8.1 PROC CORR (SAS Institute, 1996). To estimate the effects of replication, genotype and conditions, analysis of variance was carried out by SAS8.1 PROC GLM.

We performed a genome wide association study (GWAS) to detect the trait-SNP associations for all measured traits using 395,553 SNPs and the mean trait values of the 211 accessions from each of the environments. Marker-trait associations were conducted by compressed mixed linear model implemented in the Genome Association and Prediction Integrated Tool (GAPIT), a package of R software [25]. The critical *p*-value for declaring significant marker-trait association was set to 1.0 × 10^−4^. Adjacent significant SNP with distances less than 200 kb were merged into single QTL.

### Candidate gene identification for QTL affecting Fe and Zn toxicity tolerance

Here, QTL regions simultaneously meeting any of two items below were considered as important for candidate genes analysis of Fe or Zn toxicity tolerance: (1) affecting stress tolerance index for two traits; (2) consistently identified in both stress and control conditions with similar magnitude and same direction of gene effect; (3) close to previously cloned genes or mapped QTL. Besides, all QTL regions affecting concentrations of Fe and Zn in shoot were also analyzed for candidate genes. The following steps were conducted to identify candidate genes for important QTL identified. Firstly, for each important QTL region, the SNPs whose –log_10_ (p) located inside the interval of 1 unit of the peak SNP were regarded as significant. Secondly, all significant SNPs were used to check non-synonymous mutation in the coding sequence (CDS) region for all the genes located in the interval of each important QTL from the Rice Genome Annotation Project (RGAP). Thirdly, if more than two significant SNPs distributed in one gene, haplotype analysis was carried out for each of the candidate genes in each important QTL region using all non-synonymous SNPs located inside of the gene CDS region. Finally, candidate genes were determined by testing the significant differences among major haplotypes (containing more than 8 samples) for each important QTL through ANOVA.

## Results

### Phenotypic variation and trait correlation

**Fig. 1:**
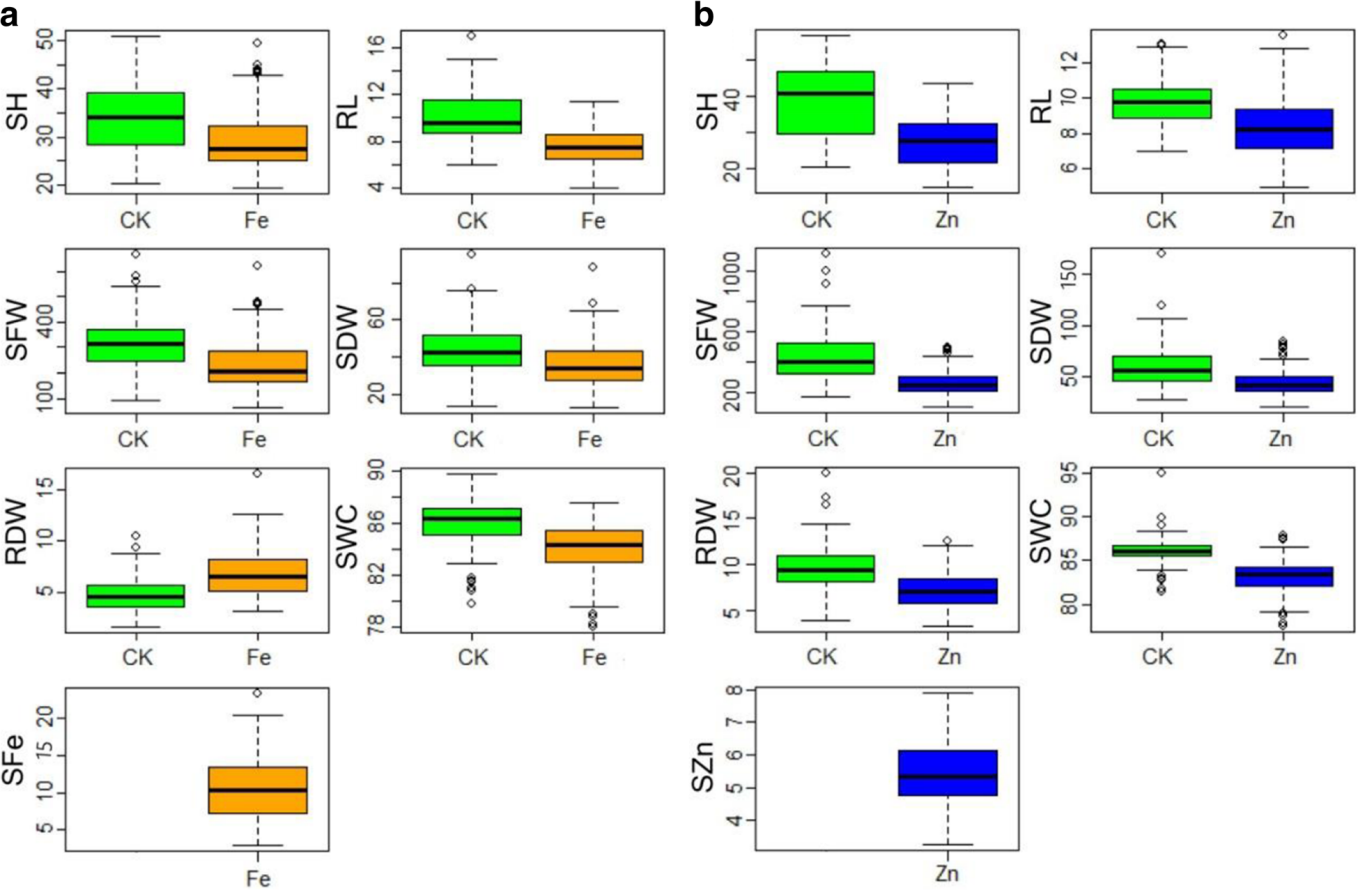
Box plot of seven measured traits in two Fe (**a**) and Zn (**b**) experiments. CK, Control condition; Fe, Ferrous iron toxicity stress condition; Zn, Zinc toxicity stress condition; SH, Shoot height; RL, Root length; SFW, Shoot fresh weight; SDW, Shoot dry weight; RDW, Root dry weight; SWC, Shoot water content; SFe, Fe concentration in shoot; SZn, Zn concentration in shoot

Wide variation of all investigated traits in both control and stress conditions were observed in the current association panel. For both Fe and Zn toxicity experiments, significant differences between control and stress conditions were observed for all traits. Compared with control, the mean values of all traits under stress conditions were considerably decreased except RDW under Fe stress condition (Fig. 1). Based on criteria of lowest SFe or SZn and highest stress tolerance index of SH, SFW and SDW, seven accessions (CC127, CC123, CC31, CC155, CC139, CC141 and CC55) were selected as Fe toxicity tolerance while eight accessions (CC101, CC120, CC55, CC109, CC83, CC218, CC123 and CC155) selected as Zn toxicity tolerance (Data not shown), and three accessions (CC55, CC123, CC155) were tolerant to both Fe and Zn toxicity stresses. ANOVA results showed that differences among genotypes and environments were highly significant for all measured traits. Genotype explained an average of 85.7% of phenotypic variance ranging from 55.9% for FeSWC to 97.3% for FeSH, and environment explained an average of 84.5% of all measured traits, ranging from 68.8% for SWC to 94.7% for SDW in Fe toxicity experiment (Additional file 2). Genotype accounted for an average of 76.9% of phenotypic variance ranging from 58.5% of Zn/CKSDW to 95.0% for CKSH, and environment explained an average of 84.6% of all traits, ranging from 75.1% for RL to 94.6% for SH in Zn toxicity experiment (Additional file 3). The trait pair-wise correlations were similar under both Fe and Zn stress conditions. The same traits among control and stress conditions had high positive correlations, suggesting the effect of iron and zinc toxicity on these traits was similar across all lines. The aboveground traits such as SH, SFW, SDW had significantly positive correlations under stress vs. control conditions, indicating that genotypic patterns of plant biomass were similar under different environments. RL under Fe stress condition had no correlation with other traits except for SFe. The SFe (SZn) had significant negative correlation with SFW, SDW, RDW and SWC under stress condition, whereby the correlation coefficients (r) were −0.61 (−0.47), −0.59 (−0.40), −0.70 (−0.57) and −0.43 (−0.40), respectively (Additional file 4).

### SNP markers

The 395,553 high quality SNP markers were evenly distributed across the 12 chromosomes covering an average 97.6% (372.96 Mb) of the rice genome published by International Rice Genome Sequencing Project [26], ranging from 94.1% (29.02 Mb) of chromosome 11 to 99.7% (28.44 Mb) of chromosome 8. The number of markers per chromosome ranged from 23,532 on chromosome 10 to 47,895 on chromosome 1. The average marker spacing was 943 bp across the whole genome ranging from 788 bp on chromosome 11 to 1041 bp on chromosome 5 (Table 1). Of these SNP markers, 23.1% were located in genes CDS region and 15.2% are non-synonymous SNPs.

**Table 1:** Distributions of markers on 12 chromosomes

Chr	Start (bp)	End (bp)	Size (Mb)	Count	Spacing	Length (Mb)^a^	Coverage (%)
1	1579	43,256,417	43.25	47,895	903	45.06	96.0
2	2057	35,935,335	35.93	40,309	891	36.82	97.6
3	20,925	36,413,109	36.39	36,812	989	37.26	97.7
4	2212	35,462,406	35.46	35,119	1010	35.86	98.9
5	10,557	29,907,310	29.90	28,729	1041	30.04	99.5
6	2922	31,246,064	31.24	31,993	977	32.12	97.3
7	1638	29,691,817	29.69	28,919	1027	30.36	97.8
8	4149	28,441,872	28.44	30,496	933	28.53	99.7
9	38,389	22,939,999	22.90	25,045	914	23.84	96.1
10	3835	23,205,372	23.20	23,532	986	23.66	98.1
11	2041	29,020,003	29.02	36,841	788	30.83	94.1
12	2372	27,530,630	27.53	29,863	922	27.76	99.2
Total			372.96	395,553	943	382.15	97.7

^a^The length of each chromosome and whole genome published by International Rice Genome Sequencing Project

### Identification of QTL associated with differentiated responses to Fe stress

**Fig. 2:**
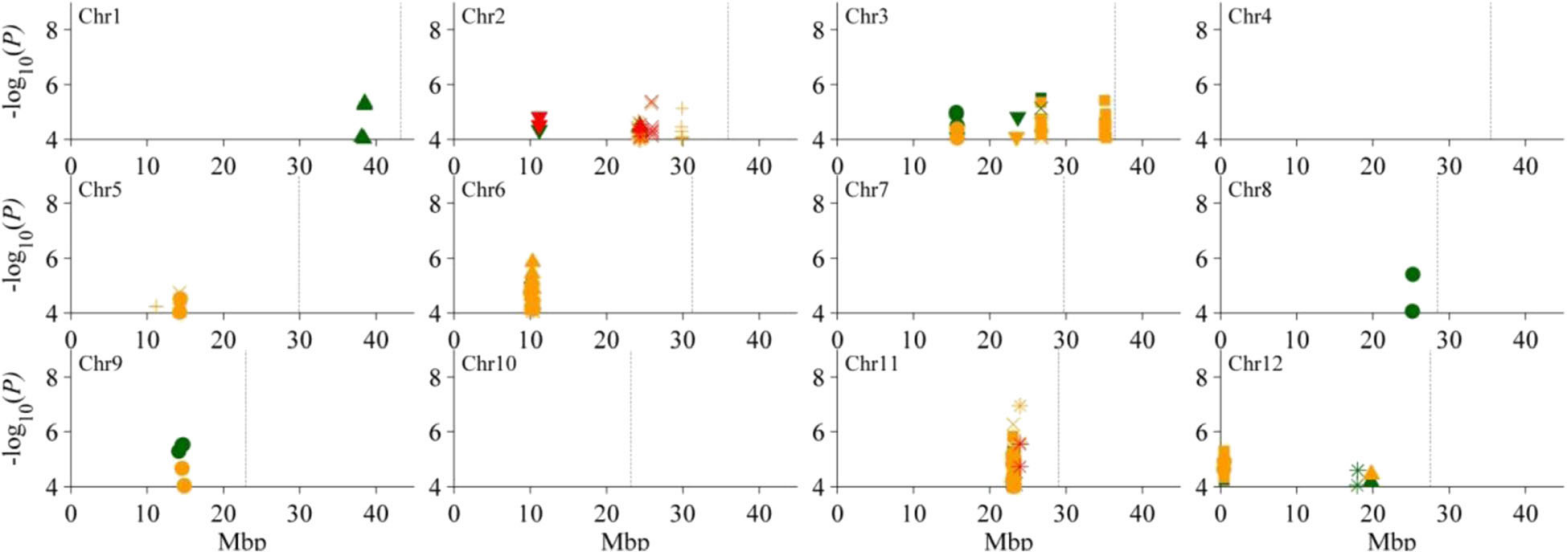
Manhattan plots of iron toxicity tolerance QTL in the whole genome. Significant SNPs from different conditions are displayed in different colors: control is green, ferrous iron stress is yellow, the ratio value between ferrous iron stress and control is red. The associated traits are represented by different symbols: shoot height = triangle up, root length = triangle down, shoot fresh weight = ×, shoot dry weight = square, root dry weight = circle, shoot water content = star, ion concentration = +

A total of 29 QTL were identified in Fe stress experiment (Additional file 5), including four detected only in control condition, 12 detected only under Fe stress condition, and 13 commonly detected under both control and Fe stress conditions. Among them, six QTL (*qSh2*, *qRl2*, *qSfw2*, *qSdw6*, *qSwc2* and *qSwc11*) were identified for the trait ratio of stress to control conditions (Fig. 2, Table 2).

**Table 2:** QTL identified with significant association to ferrous iron toxicity tolerance related traits

Trait^a^	QTL	Chr	Interval (Mb)	Control			Fe stress			Ratio of stress to control			Previously reported QTL or gene
				*P* value^b^	Effect^c^	*R* ^*2*^(%)^d^	*P* value	Effect	*R* ^*2*^(%)	*P* value	Effect	*R* ^*2*^(%)	
SH	*qSh1*	1	38.11–38.54	4.62E-06	−3.1	5.9							
	*qSh2*	2	24.35–24.56	3.07E-05	3.0	4.8	9.03E-05	2.2	6.1	3.79E-05	−0.04	7.3	
	*qSh6*	6	10.17–10.38	5.69E-06	3.1	8.3	1.38E-06	3.3	9.5				*Qsh6a* [22]
	*qSh12*	12	19.73–19.81	6.63E-05	3.2	4.4	3.49E-05	2.0	6.9				
RL	*qRl2*	2	11.17–11.19	4.59E-05	−0.7	8.3				1.51E-05	0.07	9.5	
	*qRl3*	3	23.50–23.68	1.56E-05	1.3	9.4	8.16E-05	−0.8	7.8				
SFW	*qSfw2*	2	25.87–25.97				5.32E-06	−30.1	9.7	4.21E-06	−0.05	10.2	
	*qSfw3a*	3	26.68–26.79	7.39E-06	66.1	7.0	2.82E-05	42.1	5.5				
	*qSfw5b*	5	14.23–14.33				1.83E-05	58.3	8.6				
	*qSfw6*	6	10.03–10.38				1.10E-05	40.5	9.0				
	*qSfw11*	11	23.03–23.42	8.06E-05	47.0	7.4	5.41E-07	53.5	11.9				
SDW	*qSdw3a*	3	26.68–26.79	3.16E-06	13.6	8.3	4.49E-06	12.7	9.3				*OsIRT1* [34]
	*qSdw3b*	3	35.01–35.29	2.60E-05	11.2	5.9	3.80E-06	13.6	8.8				*qSdw3* [10]
	*qSdw6*	6	10.12–10.38				1.03E-05	5.4	9.2	6.35E-05	0.06	7.9	*qSdw* [24]
	*qSdw11*	11	23.03–23.19	5.35E-06	7.1	10.3	1.51E-06	6.8	11.0				*qLBI11* [10]
	*qSdw12*	12	0.48–0.49	5.82E-05	7.3	7.9	5.24E-06	7.5	9.8				
RDW	*qRdw3*	3	15.60–15.70	1.04E-05	−0.7	8.5	4.18E-05	−0.9	7.8				*qFRRDW3* [25]
	*qRdw5b*	5	14.24–14.33				3.05E-05	1.1	8.0				
	*qRdw6*	6	10.03–10.38				1.78E-05	1.0	8.5				
	*qRdw8*	8	25.15–25.22	3.91E-06	1.0	9.4							
	*qRdw9*	9	14.56–14.84	2.97E-06	1.1	9.7	2.19E-05	1.2	8.4				*qFRRDW9–2* [25]
	*qRdw11*	11	23.00–23.18	6.87E-05	0.8	6.9	6.62E-06	1.2	9.5				
	*qRdw12*	12	0.42–0.48				1.19E-05	1.3	8.9				
SWC	*qSwc2*	2	24.17–24.41				2.38E-05	−0.8	8.4	2.79E-05	−0.01	8.6	
	*qSwc11*	11	~23.95				1.14E-07	−1.5	13.5	2.88E-06	−0.02	10.9	*qSWC* [13]
	*qSwc12*	12	17.94–17.99	2.63E-05	−1.4	9.0							
SFe	*qSFe2*	2	29.81–29.93				7.43E-06	1.7	9.4				
	*qSFe5*	5	11.18–11.19				5.66E-05	2.3	7.5				
	*qSFe6*	6	~10.38				2.62E-05	1.8	8.2				

^a^Same as in Fig. 1

^b^the peak value in the chromosome region

^c^Allele effect with respect to the minor allele

^d^Phenotypic variance explained

For SH, four QTL were identified on chromosomes 1, 2, 6 and 12. *qSh1* was only detected under the control condition and explained 5.9% of phenotypic variance. The remaining three QTL, *qSh2*, *qSh6* and *qSh12* were commonly identified under both the control and stress conditions and explained 4.4% (6.1%) to 8.3% (9.5%) of phenotypic variance in control (stress) condition. And *qSh2* also affected stress tolerance index of SH with 7.3% of phenotypic variance (Table 2).

Two QTL (*qRl2* and *qRl3*) for RL were detected on chromosomes 2 and 3. *qRl2* was identified under control condition with 8.3% of phenotypic variance, and it also affected stress tolerance index of RL and accounted for 9.5% of phenotypic variance. *qRl3* was identified under both control and stress conditions, accounting for 9.4% and 7.8% of phenotypic variance, respectively. Interestingly, minor allele at *qRl3* increased RL under control condition but reduced RL under stress condition (Table 2).

For SFW, five QTL were detected on chromosomes 2, 3, 5, 6 and 11. *qSfw2*, *qSfw5b* and *qSfw6* were identified under stress condition, explaining 8.6% to 9.7% of phenotypic variance. And *qSfw2* simultaneously affected stress tolerance index of SFW with 10.2% of phenotypic variance. *qSfw3a* and *qSfw11* were identified under both control and stress conditions and accounted for 7.0% (5.5%) and 7.4% (11.9%) of phenotypic variance in control (stress) conditions, respectively (Table 2).

Five QTL governing SDW were identified on chromosomes 3, 6, 11 and 12. *qSdw6* was detected only under stress condition and accounted for 9.2% of phenotypic variance. It also affected stress tolerance index of SDW with 7.9% of phenotypic variance. The other four QTL (*qSdw3a*, *qSdw3b*, *qSdw11* and *qSdw12*) were identified under both control and stress conditions and explained phenotypic variance ranging from 5.9% to 11.0% (Table 2).

Seven QTL affecting for RDW were identified on chromosomes 3, 5, 6, 8, 9, 11 and 12. *qRdw3*, *qRdw9* and *qRdw11* were identified under both control and stress conditions with the phenotypic variance ranging from 6.9% to 9.5%. *qRdw5b*, *qRdw6* and *qRdw12* were detected only under stress condition and explained phenotypic variance ranging from 8.0% to 8.9%. *qRdw8* was detected only under control condition with 9.4% of phenotypic variance (Table 2).

For SWC, three QTL were identified on chromosomes 2, 11 and 12. *qSwc12* was detected only under control condition and accounted for 9.0% of phenotypic variance. *qSwc2* and *qSwc11* were detected only under stress condition and explained 8.4% and 13.5% of phenotypic variance, respectively, and they also affected stress tolerance index of SWC with 8.6% and 10.9% of phenotypic variance, respectively (Table 2).

Three QTL (*qSFe2*, *qSFe5* and *qSFe6*) governing SFe were detected only under stress condition and accounted for 9.4%, 7.5% and 8.2% of phenotypic variance, respectively (Table 2).

### Identification of QTL associated with differentiated responses to Zn stress

**Fig. 3:**
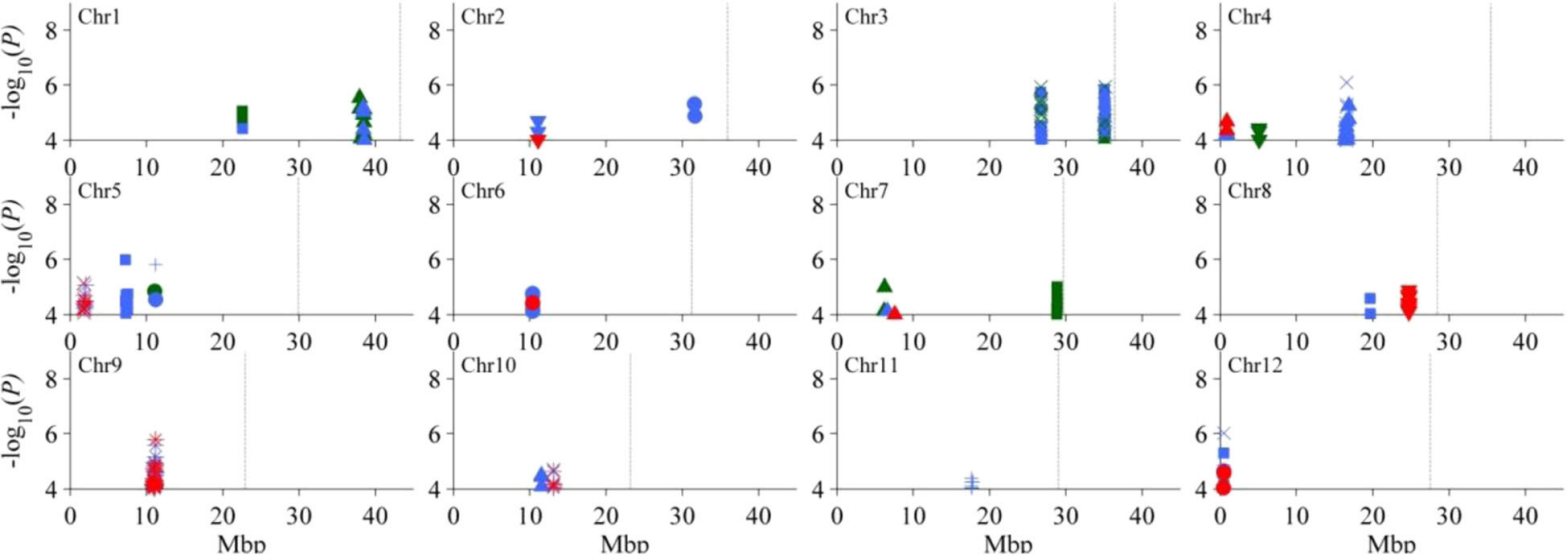
Manhattan plots of zinc toxicity tolerance QTL in the whole genome. Significant SNPs from different conditions are displayed in different colors: control is green, zinc stress is blue, the ratio between zinc stress and control is red. The symbols of associated traits are same as Fig. 2

A total of 31 QTL were identified in Zn stress experiment (Additional file 6), including three only under control condition, 19 detected only under Zn stress condition, and nine commonly detected under both stress and control conditions. Among the latter, 10 QTL (*qSh4a*, *qRl2*, *qRl8*, *qSfw5a*, *qSfw10*, *qRdw6*, *qRdw12*, *qSwc5*, *qSwc9* and *qSwc10*) had significant effects on stress tolerance index of their corresponding traits (Fig. 3, Table 3).

**Table 3:** QTL identified with significant association to zinc toxicity tolerance related traits

Trait	QTL	Chr	Interval (Mb)	Control			Zn stress			Ratio of stress to control			Previously reported
				*P* value^a^	Effect^b^	*R* ^*2*^(%)^c^	*P* value	Effect	*R* ^*2*^(%)	*P* value	Effect	*R* ^*2*^(%)	QTL
SH	*qSh1*	1	37.93–38.54	2.85E-06	−3.4	3.7	6.39E-06	−1.8	4.7				
	*qSh4a*	4	0.87–0.89				4.24E-05	1.7	3.8	2.06E-05	0.03	9.0	
	*qSh4b*	4	16.38–16.87	5.42E-05	3.0	2.7	1.70E-05	2.3	4.2				
	*qSh7*	7	6.19–6.27	1.00E-05	2.6	3.3							
	*qSh10*	10	11.54–11.55				3.22E-05	2.2	3.9				
RL	*qRl2*	2	11.10–11.11				1.99E-05	−0.6	8.0	9.73E-05	−0.05	6.9	
	*qRl4*	4	5.10–5.11	3.69E-05	0.4	6.8							
	*qRl8*	8	24.66–24.77				6.27E-05	−0.9	7.0	1.31E-05	−0.07	8.7	
SFW	*qSfw3a*	3	26.69–26.84	3.17E-06	87.7	6.7	3.91E-06	51.1	8.2				
	*qSfw3b*	3	35.03–35.16	1.15E-06	95.9	7.3	1.70E-06	61.1	8.8				*qZNT-3* [11]
	*qSfw4*	4	16.41–16.58				8.09E-07	47.1	9.4				
	*qSfw5a*	5	1.76–1.86				7.04E-05	−31.2	6.0	2.63E-05	−0.07	7.9	
	*qSfw10*	10	13.05–13.09				3.85E-05	24.6	6.4	2.05E-05	0.06	8.1	
	*qSfw12*	12	0.46–0.48				9.63E-07	64.9	9.3				
SDW	*qSdw1*	1	22.54–22.59	8.77E-06	8.7	6.4	3.80E-05	8.2	6.3				
	*qSdw3a*	3	26.69–26.84	6.20E-05	9.9	5.2	1.85E-06	8.3	4.4				
	*qSdw3b*	3	35.01–35.22	3.12E-05	9.0	5.6	1.47E-06	8.6	10.5				*qZNT-3* [11]
	*qSdw4*	4	16.46–16.58				4.13E-05	5.5	6.2				
	*qSdw5*	5	7.24–7.55				1.04E-06	6.9	9.0				
	*qSdw7*	7	28.83–28.87	9.88E-06	−7.7	6.3							
	*qSdw8*	8	19.65–19.68				2.55E-05	4.1	6.6				
	*qSdw12*	12	0.47–0.48	5.52E-05	8.6	5.2	5.11E-06	8.6	7.8				
RDW	*qRdw2*	2	31.59–31.63				4.77E-06	0.8	9.8				
	*qRdw5a*	5	11.07–11.19	1.40E-05	−1.3	8.7	2.80E-05	−1.2	8.1				
	*qRdw6*	6	~10.38				1.68E-05	−0.9	8.6	3.67E-05	−0.06	8.4	
	*qRdw12*	12	0.46–0.48				9.22E-05	−0.8	7.1	2.50E-05	−0.05	8.8	
SWC	*qSwc5*	5	1.83–2.03				8.47E-06	−1.02	9.4	3.15E-05	−0.01	7.3	
	*qSwc9*	9	10.81–11.19				2.65E-06	0.64	10.5	1.67E-06	0.01	8.6	
	*qSwc10*	10	13.05–13.12				2.31E-05	0.58	8.4	6.68E-05	0.01	6.7	
SZn	*qSZn5*	5	11.18–11.19				1.57E-06	0.72	11.8				
	*qSZn11*	11	17.63–17.83				4.10E-05	−0.48	8.5				

^a^Same as in Fig. 1

^b^the peak value in the chromosome region

^c^Allele effect with respect to the minor allele

^d^Phenotypic variance explained

Five QTL affecting SH were identified on chromosomes 1, 4, 7 and 10. *qSh7* was identified only under control condition, accounting for 3.3% of phenotypic variance. *qSh4a* and *qSh10* were detected only under stress condition and explained 3.8 and 3.9% of phenotypic variance, respectively. And *qSh4a* also affected stress tolerance index of SH with 9.0% of phenotypic variance. *qSh1* and *qSh4b* were detected under both stress and control conditions and explained 3.7% (4.7%) and 2.7% (4.2%) of phenotypic variance in control (stress) conditions, respectively (Table 3).

Three QTL affecting RL were identified on chromosomes 2, 4 and 8. *qRl2* and *qRl8* were detected under Zn stress condition and accounted for 8.0% and 7.0% of phenotypic variance, respectively. What’s more, the two QTL also affected stress tolerance index of RL with 6.9% and 8.7% of phenotypic variance, respectively. *qRl4* was identified only under control condition with 6.8% of phenotypic variance (Table 3).

For SFW, 6 QTL were detected on chromosomes 3, 4, 5, 10 and 12. Four QTL (*qSfw4*, *qSfw12*, *qSfw5a* and *qSfw10*) were identified under stress condition with phenotypic variance ranging from 6.0 to 9.4%. And the latter two QTL also affected stress tolerance index of SFW and accounted for 7.9 and 8.1%, respectively. *qSfw3a* and *qSfw3b* were detected under both control and stress conditions and explained phenotypic variation ranging from 6.7% to 8.8% (Table 3).

Eight QTL governing SDW were identified on chromosomes 1, 3, 4, 5, 7, 8 and 12. *qSdw7* was detected only under control condition, accounting for 6.3% of phenotypic variance. *qSdw4*, *qSdw5* and *qSdw8* were identified under stress condition and explained 6.2%, 9.0% and 6.6% of phenotypic variance, respectively. Four QTL (*qSdw1*, *qSdw3a*, *qSdw3b* and *qSdw12*) were detected under both control and stress conditions with phenotypic variance ranging from 4.4% to 10.5% (Table 3).

For RDW, 4 QTL were detected on chromosomes 2, 5, 6 and 12. *qRdw5a* was identified under both control and stress conditions and explained 8.7% and 8.1% of phenotypic variance, respectively. Three QTL (*qRdw2*, *qRdw6* and *qRdw12*) were detected under Zn stress condition with phenotypic variance ranging from 7.1 to 9.8%. Moreover, *qRdw6* and *qRdw12* also affected stress tolerance index of RDW with 8.4 and 8.8% of phenotypic variance, respectively (Table 3).

Three QTL (*qSwc5*, *qSwc9* and *qSwc10*) affecting SWC were identified under stress condition, accounting for 9.4%, 10.5 and 8.4% of phenotypic variance, respectively. These QTL all had significant effects on stress tolerance index of SWC and explained 7.3%, 8.6 and 6.7% of phenotypic variance, respectively (Table 3).

For SZn, two QTL (*qSZn5* and *qSZn11*) were detected under stress condition, accounting for 11.8% and 8.5% of phenotypic variance, respectively (Table 3).

### Overlap of QTL for toxicity tolerance related traits across different stress conditions

In comparison of 29 QTL detected under Fe toxicity experiment and 31 QTL detected under Zn toxicity experiment, 8 QTL (*qSh1*, *qRl2*, *qSfw3a*, *qSdw3a*, *qSdw3b*, *qSdw12*, *qRdw6* and *qRdw12*) were commonly identified in both experiments, including *qSh1* detected under control condition, *qRdw6* and *qRdw12* detected under the two stress conditions, *qSfw3a*, *qSdw3a*, *qSdw3b* and *qSdw12* detected under both control and the two stress conditions, and *qRl2* detected by stress tolerance index. In addition, *qSFe5* and *qSZn5* were colocated in the region of 11.18–11.19 Mb on chromosome 5 affecting both Fe and Zn concentrations in shoots under Fe and Zn stresses.

### Candidate genes for important QTL

**Fig. 4:**
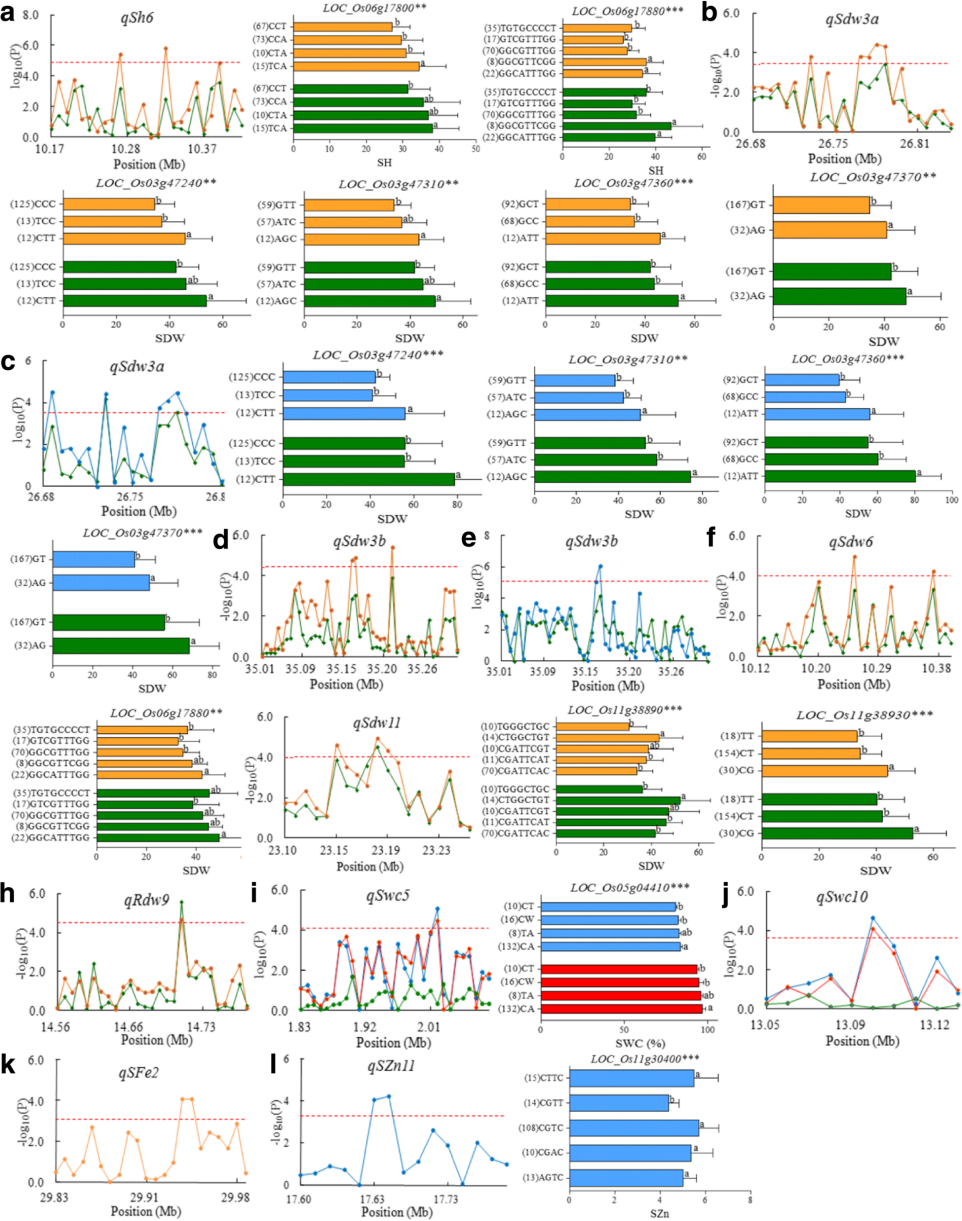
Manhattan plot of important QTL and haplotype analysis of candidate genes related to QTL including *qSh6* (**a**), *qSdw3a* (**b** and **c**), *qSdw3b* (**d** and **e**), *qSdw6* (**f**), *qSdw11* (**g**), *qRdw9* (**h**), *qSwc5* (**i**), *qSwc10* (**j**), *qSFe2* (**k**) and *qSZn*11(**l**). Each point was a gene in the region of the QTL. Line and histogram in different colors indicated different conditions: green is control condition, yellow is ferrous iron stress condition, blue is zinc stress condition and red is the ratio of zinc stress to control condition. Dash line showed the threshold to determine candidate genes. The ** and *** suggested significance of ANOVA at *p* < 0.01and *p* < 0.001, respectively. The letter on histogram (a and b) indicated multiple comparisons result at the significant level 0.01. The value in bracket was the number of individuals for each haplotype

A total of 22 candidate genes for 10 important QTL regions were found (Table 4), and only 11 candidate genes had non-synonymous SNPs within the CDS region and were performed for haplotype analysis (Fig. 4). There were 1 to 6 candidate genes for each important QTL region.

**Table 4:** List of 22 candidate genes for 10 important QTL associated with Fe or Zn toxicity tolerance

QTL	Candidate gene	Annotation
*qSh6*	*LOC_Os06g17690*	hypothetical protein
*qSh6*	*LOC_Os06g17800*	retrotransposon protein, putative, Ty3-gypsy subclass, expressed
*qSh6*	*LOC_Os06g17880*	NBS-LRR disease resistance protein, putative, expressed
*qSdw3a*	*LOC_Os03g47149*	expressed protein
*qSdw3a*	*LOC_Os03g47240*	Conserved hypothetical protein.
*qSdw3a*	*LOC_Os03g47310*	transposon protein, putative, CACTA, En/Spm sub-class, expressed
*qSdw3a*	*LOC_Os03g47330*	transposon protein, putative, CACTA, En/Spm sub-class
*qSdw3a*	*LOC_Os03g47360*	Similar to F-box domain containing protein.
*qSdw3a*	*LOC_Os03g47370*	LTPL95 - Protease inhibitor/seed storage/LTP family protein precursor, putative, expressed
*qSdw3b*	*LOC_Os03g62050*	conserved hypothetical protein
*qSdw3b*	*LOC_Os03g62060*	Similar to IAA-amino acid hydrolase 1
*qSdw3b*	*LOC_Os03g62170*	cyclase/dehydrase family protein, expressed
*qSdw6*	*LOC_Os06g17690*	hypothetical protein
*qSdw6*	*LOC_Os06g17880*	NBS-LRR disease resistance protein, putative, expressed
*qSdw11*	*LOC_Os11g38890*	retrotransposon protein, putative, unclassified, expressed
*qSdw11*	*LOC_Os11g38930*	tRNA-splicing endonuclease subunit Sen2, putative, expressed
*qSdw11*	*LOC_Os11g38959*	40S ribosomal protein S9–2, putative, expressed
*qRdw9*	*LOC_Os09g24700*	Ribosomal protein L34e domain containing protein
*qSwc5*	*LOC_Os05g04410*	peroxidase precursor, putative, expressed
*qSwc10*	*LOC_Os10g25320*	initiation factor 2 subunit family domain containing protein, expressed
*qSFe2*	*LOC_Os02g48940*	expressed protein
*qSFe2*	*LOC_Os02g48950*	ubiquitin-conjugating enzyme, putative, expressed
*qSZn11*	*LOC_Os11g30370*	OsSPL19 - SBP-box gene family member, expressed
*qSZn11*	*LOC_Os11g30400*	expressed protein

For *qSh6* in the region 10.17–10.38 Mb on chromosome 6, 102 SNPs were identified in 26 genes. Among them, three candidate genes (*LOC_Os06g17690*, *LOC_Os06g17800* and *LOC_Os06g17880*) were found for *qSh6* (Fig. 4a). Significant differences in SH were detected between different haplotypes in two candidate genes (*LOC_Os06g17800* and *LOC_Os06g17880*), and the major allele(s) in this two genes significantly decreased SH which had same effect as the peak SNP. No haplotype was found for *LOC_Os06g17690.*

For *qSdw3a* in the region 26.68–26.84 Mb on chromosome 3, 71 SNPs were identified in 25 genes, and 6 candidate genes *(LOC_Os03g47149*, *LOC_Os03g47240*, *LOC_Os03g47310*, *LOC_Os03g47330*, *LOC_Os03g47360* and *LOC_Os03g47370*) were found. Among them, *LOC_Os03g47149* was only detected under zinc stress condition (Fig. 4b and c). No haplotype was found in *LOC_Os03g47149* and *LOC_Os03g47330*. Two haplotypes were found for *LOC_Os03g47370*, and three haplotypes found for the other three genes (*LOC_Os03g47240*, *LOC_Os03g47310* and *LOC_Os03g47360*). The minor haplotypes significantly increased SDW under both Fe and Zn stress conditions.

For *qSdw3b* in the region from 35.01 to 35.29 Mb on chromosome 3, 163 SNPs were identified in 49 genes. Three candidate genes (*LOC_Os03g62050*, *LOC_Os03g62060* and *LOC_Os03g62170*) were observed for *qSdw3b*, and only *LOC_Os03g62170* was detected under Fe stress condition (Fig. 4d and e). No haplotype was found for all these genes.

For *qSdw6* in the region from 10.12 to 10.38 Mb on chromosome 6, 163 SNPs were detected in 33 genes. Two candidate genes *LOC_Os06g17690* and *LOC_Os06g17880* were found for *qSdw6* (Fig. 4f). Only *LOC_Os06g17880* had haplotype and significant phenotypic differences for SDW were observed between the five haplotypes, indicating that *LOC_Os06g17880* was the candidate gene for *qSdw6*.

For *qSdw11* in the region from 23.03 to 23.19 Mb on chromosome 11, 100 SNPs were identified in 19 genes. Three candidate genes (*LOC_Os11g38890*, *LOC_Os11g38930* and *LOC_Os11g38959*) were observed for *qSdw11*, but no haplotype was found for *LOC_Os11g38959* (Fig. 4g). Haplotype analysis revealed that major haplotype significantly decreased SDW for the two genes (*LOC_Os11g38890* and *LOC_Os11g38930*).

For *qRdw9* in the region of 14.56–14.84 Mb on chromosome 9, 84 SNPs were detected in 27 genes. Only one candidate gene *LOC_Os09g24700* was found for *qRdw9* which was associated with RDW under both control and Fe stress conditions. No haplotype was found in this gene (Fig. 4h).

For *qSwc5* in the region from 1.83 to 2.03 Mb on chromosome 5, 137 SNPs were identified in 30 genes. Only candidate gene *LOC_Os05g04410* was observed for *qSwc5*. Haplotype analysis revealed that the major haplotype increased SWC both under Zn stress condition and the Zn stress tolerance index, indicating that it was the candidate gene for *qSwc5* (Fig. 4i).

For *qSwc10* located in the region 13.05–13.12 Mb on chromosome 10, 28 SNPs were detected in 10 genes. Only one candidate gene *LOC_Os10g25320* was associated with significant SNP for *qSwc10*. No haplotype was found for the gene (Fig. 4j).

For *qSFe2* in the region from 29.81 to 29.93 Mb on chromosome 2, 58 SNPs were detected in 22 genes. *LOC_Os02g48940* and *LOC_Os02g48950* were the only two candidate genes for *qSFe2*. No haplotype was found for these two genes (Fig. 4k).

For *qSZn11* in the region 17.63–17.83 Mb on chromosome 11, 70 SNPs were identified in 15 genes. Two candidate genes *LOC_Os11g30370* and *LOC_Os11g30400* were observed for *qSZn11*. Five haplotypes were found for *LOC_Os11g30400* but no haplotype for *LOC_Os11g30370*. Haplotype CGTT had significantly lower Zn concentration in shoot than the other four haplotypes (Fig. 4l).

## Discussion

### Differential QTL expressions and their association with ion toxicity tolerance in rice

Stress tolerance in crops can be characterized in morphological, physiological and biochemical levels [27]. Many morpho-physiological traits putatively contribute to stress tolerance, and each of these traits is typically controlled by multiple genes or QTL [28]. In consideration of crop tolerance to stress is influenced by environment to a great extent, it was proposed to improve stress tolerance by marker-assisted selection (MAS) for secondary traits such as morpho-physiological traits if genes/QTL affecting the secondary traits contributing to stress tolerance could be accurately mapped and characterized [29]. Secondary traits are plant characteristics that are associated with stress tolerance under stress and should be satisfied with following tests, (1) genetically correlated with stress tolerance in the stress conditions; (2) highly heritable in the screening system; (3) enough variation among lines for the trait; (4) possible to measure the trait rapidly and economically [29]. In our study, Fe and Zn tolerance-related morphological traits (SH, RL, SFW, SDW, RDW, SWC) in stress and control conditions and physiological traits (SFe and SZn) in stress condition were measured, and their derived traits such as ratio of stress to control were also calculated and all used for input data to detect QTL. Definitely, QTL affecting ratio traits were directly related to stress tolerance [30]. However, ratio traits may reduce trait differences or variations in the population, thus probably resulting in that some QTL were undetectable. So besides comparing QTL results using ratio traits, we also compared QTL detections between stress and control conditions as we dissected drought tolerance QTL in our previous reports [31, 32].

Most of QTL at seedling stage associated with SH, RL, SFW, SDW, RDW, SWC, SFe and SZn detected in this study were specific to either Fe or Zn stress condition. Of the 29 QTL identified in Fe stress experiments, four were detected only in control condition, and 12 were detected only under Fe stress condition (Table 2). In zinc stress experiment, three were identified only in normal condition, and 19 were detected only under Zn stress condition (Table 3). Of the 13 QTL detected under both control and Fe stress conditions, one (*qRl3*) had opposite phenotypic effect under control and Fe stress conditions, three (*qSh12*, *qSfw3a* and *qSfw11*) had effects that significantly differed in magnitude under control and Fe stress conditions, and nine (*qSh2*, *qSh6*, *qSdw3a*, *qSdw3b*, *qSdw11*, *qSdw12*, *qRdw3*, *qRdw9* and *qRdw11*) behaved similarly under Fe stress and non-stress conditions. Of the 9 QTL detected under both control and Zn stress conditions, three (*qSh1*, *qSfw3a* and *qSfw3b*) had effects that differed significantly in magnitude under control and Zn stress conditions, and six (*qSh4b*, *qSdw1*, *qSdw3a*, *qSdw3b*, *qSdw12* and *qRdw5a*) behaved similarly under Zn stress and control conditions. Among the above QTL, we believe that only two types of QTL really contributed to Fe or Zn toxicity tolerance. Type I was the QTL identified by the ratio of stress to control conditions, which can reduce trait difference between stress and non-stress conditions and thus contributes to toxicity tolerance. Type II was the QTL having the same orientation and similar magnitude of phenotypic effect under both control and stress conditions, suggesting they had stable expression under stress and non-stress conditions. Therefore, a total of 14 (48.3%) different QTL contributed to Fe toxicity tolerance under Fe stress, including six QTL (*qSh2*, *qRl2*, *qSfw2*, *qSdw6*, *qSwc2* and *qSwc11*) belonged to type I and nine QTL (*qSh2*, *qSh6*, *qSdw3a*, *qSdw3b*, *qSdw11*, *qSdw12*, *qRdw3*, *qRdw9* and *qRdw11*) belonged to type II. Similarly, 16 (51.6%) QTL were associated with Zn toxicity tolerance, including 10 QTL (*qSh4a*, *qRl2*, *qRl8*, *qSfw5*, *qSfw10*, *qRdw6*, *qRdw12*, *qSwc5*, *qSwc9* and *qSwc10*) classified as type I and six (*qSh4b*, *qSdw1*, *qSdw3a*, *qSdw3b*, *qSdw12* and *qRdw5a*) QTL classified into type II. Among above QTL contributing to Fe and Zn toxicity tolerance, six QTL, including *qRl2* affecting RL, *qSdw3a*, *qSdw3b* and *qSdw12* affecting SDW and *qSFe5* (*qSZn5*) for concentrations of Fe (Zn) were detected in the same genomic regions under the both stress conditions. This indicates that there were probably some common genes or partially overlapping mechanisms for responses to Fe and Zn toxicity in rice.

### Comparison of present QTL with previously reported QTL and cloned genes

Comparison of QTL affecting Fe and Zn toxicity tolerance detected in this study with previously reported QTL or cloned genes was performed within 1 Mb physical distance. Of the 14 QTL for Fe toxicity tolerance, 8 QTL were found to locate in the same or adjacent regions with previously reported QTL or cloned genes in rice (Table 2). For example, *qSh6* affecting SH was mapped in the region 10.17–10.38 Mb on chromosome 6 which harbored previously reported *qSh6a* for SH under Fe stress condition [16]. *qSfw3a* for SFW and *qSdw3a* for SDW, detected in the region on chromosome 3 under both Fe and Zn stress conditions colocated with a previously mapped putative QTL for shoot dry weight detected by chromosomal segments substitution lines (CSSLs) [33] and *OsIRT1* (*LOC_Os03g46470*), which is a functional metal transporter of Fe and actively engaged in Fe uptake from soils [34]. Its over-expression leads to increase Fe and Zn accumulation in rice [35]. *qSdw3b* and *qSdw6* affecting SDW, located in the regions of 35.01–35.29 Mb on chromosome 3 and 10.12–10.38 Mb on chromosome 6, were colocated with *qSDW3* [8] and *qSdw* [36] for SDW, respectively. *qSdw11* affecting SDW on chromosome 11 colocated with *qLBI11* which influences leaf bronzing index under ferrous iron stress toxicity condition [8]. *qRdw3* and *qRdw9* for RDW were colocated with *qFRRDW3* and *qFRRDW9–2* for FRRDW (Fe relative root dry weight), respectively [13]. *qSwc11* affecting SWC colocated with *qSWC* for SWC [11]. Unlike QTL mapping of Fe toxicity tolerance, there are few reports on QTL mapping for Zn toxicity tolerance. Only two QTL, *qSfw3b* for SFW and *qSdw3b* for SDW in this study collocated with *qZNT-3* affecting index score of Zn toxicity at rice seedling stage [9]. QTL regions for the Fe and Zn toxicity tolerance-related traits that were identified in different mapping populations and diverse environments could be beneficial for MAS-based development of Fe and Zn toxicity tolerant rice varieties.

### Candidate gene identification for the important toxicity tolerance QTL

Although many studies reported QTL for different traits associated to Fe or Zn toxicity at seedling stage in rice [9, 13, 17, 18], no stable locus has been identified, fine-mapped or cloned so far [21]. The genetic mechanisms of rice tolerance to Fe and Zn toxicity seem to be complex. Using high density SNPs for GWAS and haplotype analysis of candidate genes, we found 22 candidate genes for 10 important QTL regions affecting the measured traits.

In the region 10.12–10.38 Mb on chromosome 6, both *qSh6* and *qSdw6* were located. Of the three candidate genes for *qSh6* and two candidate genes for *qSdw6*, the most likely one was *LOC_Os06g17880*, which encodes an NBS-LRR protein involved in disease resistance, drought tolerance and salt tolerance [37–39]. In this research, *qSdw3a* and *qSdw3b* were identified under both control and stress conditions in Fe and Zn experiments. Of the six candidate genes for *qSdw3a*, *LOC_Os03g47360* (similar to F-box domain containing protein) was the most likely one, as F-Box protein in rice was reported to be expressed under abiotic stress conditions [40]. Of the three candidate genes for *qSdw3b*, the most likely one was *LOC_Os03g62060* (similar to IAA-amino acid hydrolase 1) even though no haplotype was found in it, because it is very important for plant growth [41]. The gene *LOC_Os05g04410* is involved in peroxidase precursor whose POD-increasing activity reportedly is a defensive response to excess heavy metals in rice [42, 43]. Thus, *LOC_Os05g04410* was the most likely candidate gene for *qSwc5*. For *qSFe2*, *LOC_Os02g48950* (ubiquitin conjugating enzyme) was the most likely candidate gene, as ubiquitination plays important roles in plant abiotic stress response [44]. For *qSZn11*, *LOC_Os11g30370* encodes OsSPL19 - SBP-box, a zinc finger protein involved in a variety of biological processes [45]. Due to low density of SNP, no haplotype was found for this gene. These two genes may be both the candidate genes of *qSZn11*.

### Considerations for GWAS mapping in this study

GWAS used for association mapping of quantitative traits is becoming more and more feasible in recent years. In this study, a total of 395,553 SNPs remained after filtering low MAF (minor allele frequency) from the 700,000 SNPs, which roughly equates to 1 marker per 1 kb. It was sufficient to recognize genomic associations for Fe and Zn toxicity tolerance in rice and to identify significant SNP clusters for associated traits. The population used in this study was comparatively small and composed exclusively of *indica* accessions. Intra-subspecific analyses decrease the incidence of false-positive associations resulting from population structure. However, loci with low MAF were not detected in this study, thus some functional alleles probably escaped detection in this *indica* panel [46]. We selected significant SNPs in genes and performed haplotype analysis by non-synonymous SNP in CDS region for candidate genes analysis. There was uncertainty in candidate gene detection for some important QTL such as (*qSdw3b*, *qSFe2* and *qRdw9*) due to absence of haplotype, resulting from low numbers of significant non-synonymous SNPs. Due to the low density of SNP in some QTL regions, it was also difficult to find non-synonymous significant SNPs in CDS region in genes located in the search interval as defined in this study. For instance, the region of 11.18–11.19 Mb on chromosome 5 is important, harboring *qSFe5* and *qSZn5* for concentrations of Fe and Zn in shoots under stress conditions. However, there was no non-synonymous significant SNP in the CDS region of the gene (*LOC_Os05g19270*) within the search interval. Therefore, high SNP density and large population size are important to identify candidate genes through GWAS.

### Application in rice breeding for heavy metal toxicity tolerance

Higher Fe and Zn concentrations in the grains are desirable for human health. Ruengphayak found that the MuFRO1 mutant which tolerated Fe toxicity in the vegetative stage had 21–30% more grain Fe content than its wild type [47]. Therefore, breeding resistant rice varieties that tolerate high Fe and Zn concentrations with high content in the grains is an effective way to avoid soil pollution effects on agriculture [48]. Fe and Zn toxicity tolerance processes are difficult to define and measure. The secondary traits or symptoms associated with stress can help breeders make perform selection for stress tolerance [49]. In this study, SH, SFW and SDW were easily measured and more reliable than RL, RDW and SWC. And *SFe* (*SZn*) had significant a negative correlation with the aboveground physical traits. Consequently, favorable haplotypes or alleles of some toxicity tolerance QTL such as *qSdw3a*, *qSdw3b*, *qSdw12*, *qSFe5* and *qSZn5* may be useful for improving rice tolerance to Fe and Zn toxicity by marker-assisted selection (MAS) or QTL pyramiding. Three accessions (CC55, CC123 and CC155) with low concentrations of Fe and Zn and high tolerance index of aboveground traits under stress were identified to have strong Fe and Zn stress tolerance in this panel. At the four QTL regions mentioned above, these lines had the alleles for Fe and Zn toxicity tolerance (data not shown). So CC55, CC123 and CC155 could be used as donors in rice breeding for Fe and Zn toxicity tolerance by MAS.

## Conclusions

Large genetic variations for eight Fe and Zn toxicity tolerance related traits existed in the panel of 211 *indica* accessions. Through GWAS, a total of 14 QTL for Fe toxicity tolerance and 16 QTL for Zn toxicity tolerance were identified, respectively. *qSdw3a*, *qSdw3b*, *qSdw12* and *qSFe5* / *qSZn5* were detected in the same genomic regions under the two stress conditions, indicating that there are probably common genes and mechanisms governing Fe and Zn toxicity tolerance in rice. A total 22 candidate genes for 10 important QTL regions were determined by haplotype analyses. Five most likely candidates of five QTL (*qSh6* / *qSdw6*, *qSdw3a*, *qSdw3b*, *qSwc5*, *qSFe2*) underlying aboveground traits under stress were inferred according to functional annotation. Three accessions (CC55, CC123 and CC155) having favorable alleles at the four loci showed strong Fe and Zn stress tolerance. The candidate genes affecting Fe and Zn toxicity tolerance and tolerant accessions identified in this study provide valuable information for future functional characterization and improvement of rice varieties for heavy metal toxicity tolerance by MAS.

## Additional files

Additional file 1: Figure S1. PCA 3D plot of the first three principal components (PC) in 222 accessions (a) and 211 accessions (b); **Table S1.** List of *indica* accessions used in the present study. (DOCX 201 kb)

Additional file 2: ANOVA results of the measured traits under Fe toxicity for 211 *indica* accessions. (DOCX 25 kb)

Additional file 3: ANOVA results for the measured traits under zinc toxicity for 211 *indica* accessions. (DOCX 26 kb)

Additional file 4: Correlations between all measured traits under control (upper triangular) and stress (lower triangular) conditions in Fe (a) and Zn (b) experiments. (DOCX 275 kb)

Additional file 5: Significant SNPs associated with measured traits in Fe experiment. (XLSX 34 kb)

Additional file 6: Significant SNPs associated with measured traits in Zn experiment. (XLSX 40 kb)

## Abbreviations

CK: Control condition
Fe/CK: Ratio of ferrous iron stress to control condition
MAS: Marker-assisted selection
RDW: Root dry weight
RL: Root length
SDW: Shoot dry weight
SFe: Fe concentration in shoot;
SFW: Shoot fresh weight
SH: Shoot height
SWC: Shoot water content
SZn: Zn concentration in shoot;
Zn/CK: Ratio of zinc stress to control condition
