# Supplementary material for: QTL mapping and candidate gene analysis of ferrous iron and zinc toxicity tolerance at seedling stage in rice by genome-wide association study
Source: BMC Genomics. 2017 Oct 27;18:828. doi: 10.1186/s12864-017-4221-5 (PMC5658907; doi:10.1186/s12864-017-4221-5)
Supplement: Supplementary file 1 — PCA 3D plot of the first three principal components (PC) in 222 accessions (a) and 211 accessions (b); Table S1. List of indica accessions used in the present study. (DOCX 201 kb) [file 12864_2017_4221_MOESM1_ESM.docx]

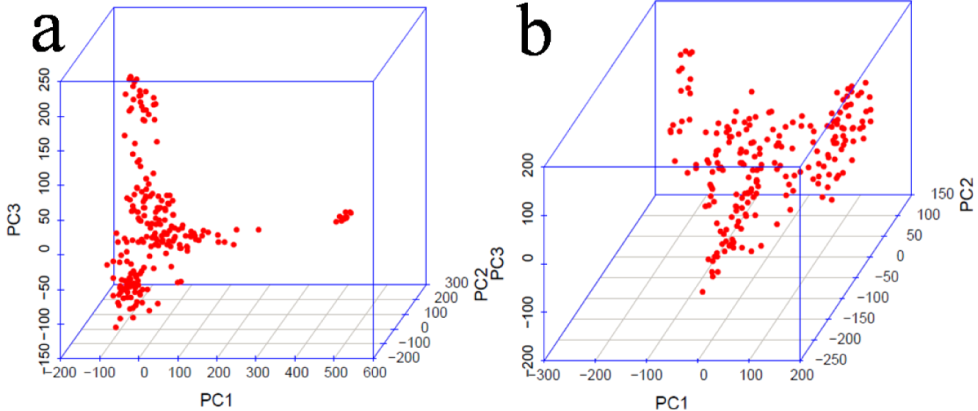


**Additional file 1: Figure S1.** PCA 3D plot of the first three principal components (PC) in 222 accessions (a) and 211 accessions (b)

**Additional file 1: Table S1.** List of *indica* accessions used in the present study

| Group | Code | IRGC Acc. No. | DESIGNATION | Subpopulation | ORIGIN |
| --- | --- | --- | --- | --- | --- |
| group1 | CC1 | 117460 | DA 9::IRGC 5854-1 | indica | Bangladesh |
| group1 | CC2 | 122287 | WAS 198-B-3-1-3::C1 | Oryza sativa (Linnaeus) | Unknown |
| group1 | CC4 | 120982 | IR 32453-20-3-2-2::IRGC 76331-1 | indica | Philippines |
| group1 | CC5 | 122290 | WAS 206-B-B-2-2-1::C1 | Oryza sativa (Linnaeus) | Unknown |
| group1 | CC6 | 122140 | KOGONI 91-1::C1 | Oryza sativa (Linnaeus) | Unknown |
| group1 | CC7 | 121855 | WAS 208-B-B-5-1-1-3::C1 | indica | Unknown |
| group1 | CC8 | 122285 | WAS 173-B-B-6-2-2::C1 | Oryza sativa (Linnaeus) | Unknown |
| group1 | CC9 | 122232 | SAHEL 108::C1 | Oryza sativa (Linnaeus) | Unknown |
| group1 | CC10 | 122088 | IR 1561-228-3-3::IRGC 32627-C1 | Oryza sativa (Linnaeus) | Philippines |
| group1 | CC11 | 122089 | IR 19746-28-2-2::IRGC 78072-C1 | Oryza sativa (Linnaeus) | Philippines |
| group1 | CC12 | 121753 | IR 60::IRGC 63493-C1 | indica | Philippines |
| group1 | CC13 | 121748 | IR 2006-P12-12-2::IRGC 32675-C1 | indica | Philippines |
| group1 | CC14 | 122091 | IR 22::IRGC 11356-C1 | Oryza sativa (Linnaeus) | Philippines |
| group1 | CC16 | 117280 | ZHENSHAN 97 B | Oryza sativa (Linnaeus) | China |
| group1 | CC17 | 120983 | IR 43::IRGC 117005-1 | indica | Philippines |
| group1 | CC18 | 121760 | IR 72::C1 | indica | Philippines |
| group1 | CC20 | 120981 | IR 31917-45-3-2::IRGC 78132-1 | indica | Philippines |
| group1 | CC21 | 121854 | WAS 207-B-B-3-1-1::C1 | indica | Unknown |
| group1 | CC22 | 120893 | BG 34-11::IRGC 15782-1 | indica | Sri Lanka |
| group1 | CC23 | 121139 | TNAU 7456::IRGC 39858-1 | indica | India |
| group1 | CC24 | 117757 | IR 36 | Oryza sativa (Linnaeus) | Philippines |
| group1 | CC25 | 122239 | SAMBALA MALO::C1 | Oryza sativa (Linnaeus) | Unknown |
| group1 | CC26 | 122042 | ELONI::C1 | Oryza sativa (Linnaeus) | Unknown |
| group1 | CC27 | 117758 | IR 8 | Oryza sativa (Linnaeus) | Philippines |
| group1 | CC29 | 121042 | LONG ZI 1::IRGC 63726-1 | indica | China |
| group1 | CC30 | 121147 | UP 1537::IRGC 70490-1 | indica | Colombia |
| group1 | CC31 | 121771 | JAMAJIGI::C1 | indica | Unknown |
| group1 | CC32 | 121857 | WAS 30-11-4-6-2-2-1::C1 | indica | Unknown |
| group1 | CC33 | 117659 | BINULAWAN | Oryza sativa (Linnaeus) | Philippines |
| group1 | CC34 | 122255 | SONA::IRGC 26971-C1 | Oryza sativa (Linnaeus) | India |
| group1 | CC36 | 120861 | AI LAN KE 1110::IRGC 67034-1 | indica | China |
| group1 | CC37 | 121799 | NIONOKA::C1 | indica | Unknown |
| group1 | CC38 | 117271 | MINGHUI 63 | Oryza sativa (Linnaeus) | China |
| group1 | CC39 | 121827 | TELIMANI::C1 | indica | Unknown |
| group1 | CC40 | 120972 | HONG ZUI ZAO::IRGC 68090-1 | indica | China |
| group1 | CC41 | 122297 | WAS 63-22-5-9-10-1::C1 | Oryza sativa (Linnaeus) | Unknown |
| group1 | CC43 | 121853 | WAS 202-B-B-1-1-2::C1 | indica | Unknown |
| group1 | CC44 | 122289 | WAS 203-B-B-2-4-1::C1 | Oryza sativa (Linnaeus) | Unknown |
| group1 | CC45 | 117268 | IR 64-21 | Oryza sativa (Linnaeus) | Philippines |
| group1 | CC48 | 121751 | IR 52::IRGC 53434-C1 | indica | Philippines |
| group1 | CC50 | 121847 | WAS 181-B-6-3::C1 | indica | Unknown |
| group1 | CC51 | 120977 | ICTA POLOCHIC::IRGC 116997-1 | indica | Guatemala |
| group1 | CC52 | 117829 | ORYZICA LLANOS 5 | Oryza sativa (Linnaeus) | Colombia |
| group1 | CC54 | 117912 | TEQING | Oryza sativa (Linnaeus) | China |
| group1 | CC55 | 120863 | ANAYANSI::IRGC 77474-1 | indica | Colombia |
| group1 | CC56 | 120916 | CIMARRON::IRGC 116967-1 | indica | Venezuela |
| group1 | CC62 | 120947 | E ZI 124::IRGC 70215-1 | indica | China |
| group1 | CC63 | 122090 | IR 20::C1 | Oryza sativa (Linnaeus) | Philippines |
| group1 | CC66 | 121850 | WAS 194-B-3-2-5::C1 | indica | Unknown |
| group1 | CC67 | 121089 | PSBRC 18::IRGC 117375-1 | indica | Philippines |
| group1 | CC68 | 121851 | WAS 197-B-6-3-11::C1 | indica | Unknown |
| group1 | CC69 | 120988 | IR 77384-12-35-3-12-1-B::IRGC 117299-1 | indica | Philippines |
| group1 | CC71 | 120939 | DJOGOLON DJOGOLON::IRGC 75577-1 | indica | Upper Volta |
| group1 | CC73 | 122011 | CICA 8::C1 | Oryza sativa (Linnaeus) | Unknown |
| group1 | CC76 | 121889 | PELITA JANGGUT::IRGC 43540-1 | indica | Indonesia |
| group1 | CC77 | 122292 | WAS 33-B-B-15-1-4-5::C1 | Oryza sativa (Linnaeus) | Unknown |
| group1 | CC78 | 120979 | INIAP 415::IRGC 117001-1 | indica | Ecuador |
| group1 | CC79 | 122291 | WAS 21-B-B-20-4-3-3::C1 | Oryza sativa (Linnaeus) | Unknown |
| group1 | CC80 | 121163 | ZAO SHOU 691-11::IRGC 70447-1 | indica | China |
| group1 | CC81 | 122284 | WAS 170-B-B-1-1::C1 | Oryza sativa (Linnaeus) | Unknown |
| group1 | CC83 | 121846 | WAS 169-B-B-4-2-1::C1 | indica | Unknown |
| group1 | CC84 | 117454 | CO 39::IRGC 51231-1 | indica | India |
| group1 | CC85 | 117907 | TAICHUNG NATIVE 1 | Oryza sativa (Linnaeus) | Taiwan |
| group1 | CC86 | 121053 | MG 2::IRGC 79837-1 | indica | Brazil |
| group1 | CC87 | 122288 | WAS 199-B-1-2-1::C1 | Oryza sativa (Linnaeus) | Unknown |
| group1 | CC88 | 122112 | IR 74371-54-1-1::C1 | Oryza sativa (Linnaeus) | Philippines |
| group1 | CC89 | 121689 | BADKALAMKATI::IRGC 45011-1 | indica | India |
| group1 | CC91 | 122093 | IR 2344-P1 PB-9-3-2B::IRGC 39317-C1 | Oryza sativa (Linnaeus) | Philippines |
| group1 | CC92 | 122156 | MACAN BINUNDOK::IRGC 8245-C1 | Oryza sativa (Linnaeus) | Philippines |
| group1 | CC93 | 121738 | GAMBIAKA KOKOUM::C1 | indica | Unknown |
| group1 | CC94 | 121752 | IR 55419-04::C1 | indica | Philippines |
| group1 | CC95 | 121705 | APO::C1 | indica | Philippines |
| group1 | CC96 | 121044 | MA GU ZI HE::IRGC 68212-1 | indica | China |
| group1 | CC97 | 121639 | SAO::IRGC 61467-1 | admixed | Liberia |
| group1 | CC98 | 121162 | ZAO SHAO ZHAN::IRGC 68318-1 | indica | China |
| group1 | CC100 | 121024 | KIRIMURUNGA::IRGC 15585-1 | indica | Sri Lanka |
| group1 | CC101 | 121034 | LAL BAGDAR::IRGC 77272-1 | indica | Bangladesh |
| group1 | CC102 | 122099 | IR 57924-24::IRTP 16675-C1 | Oryza sativa (Linnaeus) | Philippines |
| group1 | CC105 | 120964 | HAO HOM::IRGC 12931-1 | indica | Laos |
| group1 | CC108 | 117623 | BR24 | Oryza sativa (Linnaeus) | Bangladesh |
| group1 | CC109 | 120879 | BADA DHAN::IRGC 26540-1 | indica | Bangladesh |
| group1 | CC110 | 122206 | POKKALI::IRGC 108921-C1 | Oryza sativa (Linnaeus) | India |
| group1 | CC111 | 122298 | WAY RAREM::IRTP 23013-C1 | Oryza sativa (Linnaeus) | Unknown |
| group1 | CC113 | 121835 | UPL RI 5::IRTP 7034-C1 | indica | Philippines |
| group1 | CC114 | 121591 | IR 5::IRGC 10321-1 | indica | Philippines |
| group1 | CC115 | 121774 | KALINGA III::C1 | indica | India |
| group1 | CC116 | 122025 | CT 6510-24-1-2::C1 | Oryza sativa (Linnaeus) | Unknown |
| group1 | CC119 | 122076 | H 15-23-DA::C1 | Oryza sativa (Linnaeus) | Unknown |
| group1 | CC120 | 120892 | BG 301::IRGC 117315-1 | indica | Sri Lanka |
| group1 | CC121 | 120984 | IR 59469-2B-3-2::IRGC 99703-1 | indica | Philippines |
| group1 | CC122 | 122029 | DANAU LAUT TAWAR::C1 | Oryza sativa (Linnaeus) | Unknown |
| group1 | CC123 | 122272 | UPL RI 7::IRTP 9897-C1 | Oryza sativa (Linnaeus) | Philippines |
| group1 | CC125 | 121642 | SOM CAU 70 A::IRGC 8227-1 | indica | Viet Nam |
| group1 | CC126 | 121136 | TELOVOLANA::IRGC 69969-1 | admixed-indica | Madagascar |
| group1 | CC127 | 121159 | YE TI ZHAN::IRGC 68296-1 | indica | China |
| group1 | CC128 | 121881 | JC 120::IRGC 9178-1 | indica | India |
| group1 | CC135 | 122179 | NAM SA GUI 19::IRTP 6892-C1 | Oryza sativa (Linnaeus) | Thailand |
| group1 | CC136 | 122181 | NONA BOKRA::IRGC 22710-C1 | Oryza sativa (Linnaeus) | India |
| group1 | CC138 | 121062 | NAKABAWA::IRGC 70676-1 | indica | Kenya |
| group1 | CC139 | 121151 | VARY VATO MENAHODITRA::IRGC 69111-1 | Oryza sativa (Linnaeus) | Madagascar |
| group1 | CC140 | 120913 | CHORUA KARTIKSAIL::IRGC 77230-1 | indica | Bangladesh |
| group1 | CC141 | 121035 | LALBAJAM::IRGC 49227-1 | indica | Bangladesh |
| group1 | CC142 | 121137 | TI KU::IRGC 1224-1 | indica | China |
| group1 | CC143 | 121725 | CHERIVIRUPPU::IRGC 19928-C1 | indica | India |
| group1 | CC144 | 121095 | RAJ BHOG::IRGC 77294-1 | indica | Bangladesh |
| group1 | CC145 | 122098 | IR 57920-AC 25-2-B::C1 | Oryza sativa (Linnaeus) | Philippines |
| group1 | CC147 | 117276 | SADU CHO | Oryza sativa (Linnaeus) | Korea Rep |
| group1 | CC148 | 117915 | TKM 6 | Oryza sativa (Linnaeus) | India |
| group1 | CC150 | 121011 | KARAYAL::IRGC 51001-1 | indica | Sri Lanka |
| group1 | CC151 | 120952 | Ea houm::IRGC 12925-1 | indica | Laos |
| group1 | CC153 | 117501 | JC 92::IRGC 9176-1 | indica | India |
| group1 | CC155 | 120970 | HONG MI DONG MAO ZHAN::IRGC 68078-1 | indica | China |
| group1 | CC156 | 117574 | RTS 4::IRGC 8177-1 | indica | Viet Nam |
| group1 | CC158 | 121094 | RACE::IRGC 15706-1 | indica | Sri Lanka |
| group1 | CC161 | 117841 | PAPPAKU | Oryza sativa (Linnaeus) | Taiwan |
| group1 | CC163 | 121156 | XI GU HONG::IRGC 74226-1 | indica | China |
| group1 | CC164 | 121019 | KHAO DAW TAI::IRGC 24108-1 | indica | Thailand |
| group1 | CC167 | 121074 | NS 1611::IRGC 68963-1 | indica | Madagascar |
| group1 | CC168 | 117447 | CHITRAJ (DA 23)::IRGC 6208-1 | indica | Bangladesh |
| group1 | CC169 | 120862 | AN QING ZAO::IRGC 72577-1 | indica | China |
| group1 | CC170 | 121087 | PINURSIGI::IRGC 26889-1 | indica | Philippines |
| group1 | CC171 | 121080 | PAWHTUN::IRGC 33562-1 | indica | Myanmar |
| group1 | CC172 | 121049 | MAKALIOKA::IRGC 77864-1 | indica | Madagascar |
| group1 | CC173 | 121066 | NCS 130::IRGC 51879-1 | indica | India |
| group1 | CC174 | 121118 | SHONTH::IRGC 74717-1 | indica | India |
| group1 | CC175 | 117622 | RTS 14 | Oryza sativa (Linnaeus) | Viet Nam |
| group1 | CC176 | 121064 | NARGUNI::IRGC 74713-1 | indica | India |
| group1 | CC177 | 122236 | SALUMPIKIT::IRTP 4777-C1 | Oryza sativa (Linnaeus) | Philippines |
| group1 | CC180 | 120909 | CHI TOU HUANG 1::IRGC 51280-1 | indica | China |
| group1 | CC182 | 121124 | SOMIMADAMO::IRGC 69044-1 | indica | Madagascar |
| group1 | CC183 | 120880 | BAI HE::IRGC 76437-1 | indica | China |
| group1 | CC184 | 117636 | AI-CHIAO-HONG | Oryza sativa (Linnaeus) | China |
| group1 | CC185 | 117681 | CHANG CH'SANG HSU TAO | Oryza sativa (Linnaeus) | China |
| group1 | CC186 | 121023 | KINANDANG PUTI::IRGC 44513-1 | indica | Philippines |
| group1 | CC188 | 121157 | XI NUO ZAO::IRGC 68279-1 | indica | China |
| group1 | CC189 | 121045 | MA WAINE OHN::IRGC 33357-1 | indica | Myanmar |
| group1 | CC190 | 121099 | RAY JAZAYKAYZ::IRGC 62181-1 | indica | Bhutan |
| group1 | CC191 | 121063 | NAN TE 113::IRGC 70345-1 | indica | China |
| group1 | CC193 | 122000 | CARREON::IRGC 32575-C1 | Oryza sativa (Linnaeus) | Philippines |
| group1 | CC195 | 117564 | PTB 9::IRGC 6274-1 | indica | India |
| group1 | CC197 | 121172 | MAHADETHE::IRGC 74762-1 | indica | India |
| group1 | CC198 | 121105 | RUZZ (HABUR)::IRGC 55679-1 | indica | Iran |
| group1 | CC199 | 121073 | NS 113::IRGC 68838-1 | admixed-indica | Madagascar |
| group1 | CC200 | 121237 | DA 29 (SR 26 B)::IRGC 25850-1 | indica | Bangladesh |
| group1 | CC201 | 117784 | KIANG-CHOU-CHIU | Oryza sativa (Linnaeus) | Taiwan |
| group1 | CC202 | 117525 | MADAEL::IRGC 7722-1 | indica | Sri Lanka |
| group1 | CC203 | 121085 | PICONEGRO::IRGC 117022-1 | indica | Ecuador |
| group1 | CC205 | 121010 | KANNI MURUNGA::IRGC 15432-1 | indica | Sri Lanka |
| group1 | CC206 | 121092 | QING GU::IRGC 59839-1 | indica | China |
| group1 | CC207 | 121161 | ZALCHA::IRGC 62190-1 | indica | Bhutan |
| group1 | CC208 | 121104 | RTS 5::IRGC 8233-1 | indica | Viet Nam |
| group1 | CC209 | 121020 | KHAO PON::IRGC 48114-1 | indica | Thailand |
| group1 | CC210 | 121785 | LOHAMBITRO 224::GERVEX 5144-C1 | indica | Unknown |
| group1 | CC211 | 121015 | KATI::IRGC 67838-1 | indica | Bhutan |
| group1 | CC212 | 117587 | TD 25::IRGC 9146-1 | Oryza sativa (Linnaeus) | Thailand |
| group1 | CC213 | 121823 | SOMIZY::GERVEX 8419-C1 | indica | Unknown |
| group1 | CC215 | 117521 | LAL AMAN::IRGC 46202-1 | indica | India |
| group1 | CC216 | 121145 | TUNG CH'IU AI::IRGC 34265-1 | aus | China |
| group1 | CC217 | 120914 | CHUA DAU::IRGC 4785-1 | indica | China |
| group1 | CC218 | 117682 | CHAU | Oryza sativa (Linnaeus) | Viet Nam |
| group1 | CC219 | 117533 | MTU 9::IRGC 7919-1 | indica | India |
| group1 | CC220 | 117826 | O-LUEN-CHEUNG | Oryza sativa (Linnaeus) | Taiwan |
| group1 | CC221 | 122159 | MAMORIAKA 114::GERVEX 5176-C1 | Oryza sativa (Linnaeus) | Unknown |
| group1 | CC224 | 121131 | SUTHUWEE::IRGC 8915-1 | indica | Sri Lanka |
| group1 | CC225 | 121110 | SAN DU BAI MI HONG GU::IRGC 59849-1 | indica | China |
| group1 | CC226 | 121106 | SADA DANGA BORO::IRGC 77298-1 | indica | Bangladesh |
| group1 | CC227 | 121965 | ARANG::IRGC 43322-C1 | Oryza sativa (Linnaeus) | Indonesia |
| group1 | CC228 | 121420 | MAHSURI::IRGC 10929-1 | indica | Malaysia |
| group1 | CC231 | 121070 | NORUNKAN::IRGC 8934-1 | indica | Sri Lanka |
| group1 | CC232 | 117478 | GIE 57::IRGC 8231-1 | indica | Viet Nam |
| group1 | CC233 | 121098 | RATHKANDIRAM::IRGC 36507-1 | indica | Sri Lanka |
| group1 | CC235 | 121164 | ZI GAN NAN GU::IRGC 70468-1 | indica | China |
| group1 | CC236 | 121165 | ZS 4::IRGC 56707-1 | indica | Zambia |
| group1 | CC237 | 121663 | GAJPATI::IRGC 58981-1 | indica | Nepal |
| group1 | CC238 | 121146 | UBA MURALI::IRGC 25928-1 | indica | Bangladesh |
| group1 | CC239 | 121158 | YAKADA::IRGC 51096-1 | indica | Sri Lanka |
| group1 | CC240 | 121176 | XI GU ZAO::IRGC 72360-1 | indica | China |
| group1 | CC241 | 121028 | KUMBI::IRGC 752-1 | indica | China |
| group1 | CC242 | 121059 | MOTTA SAMBA::IRGC 36489-1 | indica | Sri Lanka |
| group1 | CC243 | 117470 | FANDRAPOTSY::IRGC 10984-1 | indica | Madagascar |
| group1 | CC246 | 120895 | BIRAIN 360::IRGC 6550-1 | indica | Bangladesh |
| group1 | CC247 | 121077 | PANAKALI::IRGC 47399-1 | indica | Sri Lanka |
| group1 | CC249 | 121140 | TSIAMPOETRY::IRGC 77902-1 | indica | Madagascar |
| group1 | CC250 | 121225 | THAPACHINIYA::IRGC 16234-1 | indica | Nepal |
| group1 | CC254 | 121122 | SOKOU MALSIRA::IRGC 77301-1 | indica | Bangladesh |
| group1 | CC255 | 121811 | ROJOFOTSY 693::GERVEX 8407-C1 | admixed-indica | Unknown |
| group1 | CC256 | 117559 | PETA::IRGC 32571-1 | indica | Indonesia |
| group1 | CC257 | 121041 | LOKU SAMBA::IRGC 31462-1 | indica | Sri Lanka |
| group1 | CC258 | 117567 | RATHUWEE::IRGC 8952-1 | indica | Sri Lanka |
| group1 | CC260 | 117849 | PEH-KUH-TSAO-TU | Oryza sativa (Linnaeus) | Taiwan |
| group1 | CC261 | 121091 | PURBIA (KALANSAR)::IRGC 59189-1 | indica | Nepal |
| group1 | CC264 | 121990 | BOTRA MAITSO::GERVEX 8389-C1 | Oryza sativa (Linnaeus) | Unknown |
| group1 | CC265 | 117691 | CO 18 | Oryza sativa (Linnaeus) | India |
| group1 | CC266 | 121083 | PERUM KARUPPAN::IRGC 15524-1 | indica | Sri Lanka |
| group1 | CC267 | 120856 | 17/79/02-005::IRGC 51080-1 | indica | Sri Lanka |
| group1 | CC268 | 120924 | DA 5::IRGC 5855-1 | indica | Bangladesh |
| group1 | CC269 | 121963 | ANGIFOTSY 685::GERVEX 4717-C1 | Oryza sativa (Linnaeus) | Unknown |
| group1 | CC270 | 121142 | TSIPALA MENA::IRGC 69977-1 | indica | Madagascar |
| group1 | CC272 | 120959 | GOPAL::IRGC 61953-1 | indica | Nepal |
| group1 | CC273 | 117848 | PEH-KUH | Oryza sativa (Linnaeus) | Taiwan |
| group1 | CC274 | 121834 | TSIPALA FOTSY 1883::GERVEX 5387-C1 | indica | Unknown |
| group1 | CC275 | 121717 | BODOMANO::GERVEX 8343-C1 | admixed-indica | Unknown |
| group1 | CC276 | 117880 | SERATOES HARI | Oryza sativa (Linnaeus) | Indonesia |
| group1 | CC279 | 121839 | VARY VATO 154::GERVEX 5429-C1 | indica | Unknown |
| group1 | CC280 | 117840 | PAO TOU HUNG | Oryza sativa (Linnaeus) | China |
| group1 | CC281 | 121829 | TOKAMBANY 663::GERVEX 8358-C1 | indica | Unknown |
| group1 | CC282 | 121708 | ARC 15872::IRGC 43249-C1 | indica | India |
| group1 | CC283 | 120904 | BYAT KYAR::IRGC 33004-1 | indica | Myanmar |
| group1 | CC284 | 117520 | LAGEADO::IRGC 50490-1 | indica | Brazil |
| group1 | CC286 | 121830 | TOKAMBANY 669::GERVEX 8406-C1 | admixed-indica | Unknown |
| group1 | CC290 | 121812 | ROJOMENA 1034::GERVEX 8412-C1 | admixed-indica | Unknown |
| group2 | CC117 | 121632 | ORYZICA SABANA 10::IRGC 117018-1 | Oryza sativa (Linnaeus) | Colombia |
| group2 | CC160 | 122096 | IR 53236-275-1::C1 | Oryza sativa (Linnaeus) | Philippines |
| group2 | CC166 | 121804 | PCT 11\0\0\2,BO 1>55-1-3-1::C1 | tropical-japonica | Unknown |
| group2 | CC178 | 121807 | PEH PI NUO::IRGC 8266-C1 | admixed-japonica | China |
| group2 | CC229 | 121794 | MANGAVAVA FOTSILANSTSIKA 1177::GERVEX 5719-C1 | tropical-japonica | Unknown |
| group2 | CC245 | 122178 | NAM ROO::C1 | Oryza sativa (Linnaeus) | Unknown |
| group2 | CC248 | 121722 | C 21::IRGC 331-C1 | tropical-japonica | Philippines |
| group2 | CC262 | 122080 | IAC 165::GERVEX 8508-C1 | Oryza sativa (Linnaeus) | Unknown |
| group2 | CC61 | 117560 | PIN KAEO::IRGC 5803-1 | tropical-japonica | Thailand |
| group2 | CC72 | 121844 | WAB 706-3-4-K4-KB-1::C1 | tropical-japonica | Unknown |
| group2 | CC82 | 121701 | 91-385::IRGC 63466-C1 | temperate-japonica | Bhutan |
