# Supplementary material for: QTL mapping and candidate gene analysis of ferrous iron and zinc toxicity tolerance at seedling stage in rice by genome-wide association study
Source: BMC Genomics. 2017 Oct 27;18:828. doi: 10.1186/s12864-017-4221-5 (PMC5658907; doi:10.1186/s12864-017-4221-5)
Supplement: Supplementary file 2 — ANOVA results of the measured traits under Fe toxicity for 211 indica accessions. (DOCX 25 kb) [file 12864_2017_4221_MOESM2_ESM.docx]

**Additional file 3**

ANOVA results of all the measured traits under Fe toxicity for 211 *indica* accessions

| Trait | Source of variation | df | SS | MS | F | P- value | R^2^ (%) |
| --- | --- | --- | --- | --- | --- | --- | --- |
| CKSH | Genotype | 207 | 20089.19 | 97.05 | 27.8 | <.0001 | 96.5 |
|  | Rep | 1 | 15.68 | 15.68 | 4.5 | 0.0353 |  |
|  | Error | 207 | 723.47 | 3.50 |  |  |  |
| CKRL | Genotype | 207 | 1005.83 | 4.86 | 3.5 | <.0001 | 77.9 |
|  | Rep | 1 | 0.27 | 0.27 | 0.2 | 0.6581 |  |
|  | Error | 207 | 285.63 | 1.38 |  |  |  |
| CKSFW | Genotype | 207 | 3261212.44 | 15754.65 | 17.1 | <.0001 | 94.0 |
|  | Rep | 1 | 15977.74 | 15977.74 | 17.3 | <.0001 |  |
|  | Error | 207 | 190976.98 | 922.59 |  |  |  |
| CKSDW | Genotype | 207 | 58211.14 | 281.21 | 18.4 | <.0001 | 94.6 |
|  | Rep | 1 | 158.71 | 158.71 | 10.4 | 0.0015 |  |
|  | Error | 207 | 3172.33 | 15.33 |  |  |  |
| CKRDW | Genotype | 207 | 1025.48 | 4.95 | 20.9 | <.0001 | 95.3 |
|  | Rep | 1 | 0.90 | 0.90 | 3.8 | 0.0525 |  |
|  | Error | 207 | 49.19 | 0.24 |  |  |  |
| CKSWC | Genotype | 207 | 577.44 | 2.79 | 2.3 | <.0001 | 69.9 |
|  | Rep | 1 | 1.00 | 1.00 | 0.8 | 0.3616 |  |
|  | Error | 207 | 247.16 | 1.19 |  |  |  |
| FeSH | Genotype | 207 | 14691.83 | 70.98 | 36.3 | <.0001 | 97.3 |
|  | Rep | 1 | 1.00 | 1.00 | 0.5 | 0.4753 |  |
|  | Error | 206 | 403.05 | 1.96 |  |  |  |
| FeRL | Genotype | 207 | 1219.44 | 5.89 | 5.1 | <.0001 | 83.6 |
|  | Rep | 1 | 0.01 | 0.01 | 0.0 | 0.9308 |  |
|  | Error | 206 | 239.28 | 1.16 |  |  |  |
| FeSFW | Genotype | 207 | 3266675.08 | 15781.04 | 33.8 | <.0001 | 96.8 |
|  | Rep | 1 | 12215.47 | 12215.47 | 26.2 | <.0001 |  |
|  | Error | 207 | 96686.18 | 467.08 |  |  |  |
| FeSDW | Genotype | 207 | 56389.65 | 272.41 | 29.5 | <.0001 | 96.5 |
|  | Rep | 1 | 159.86 | 159.86 | 17.3 | <.0001 |  |
|  | Error | 207 | 1910.16 | 9.23 |  |  |  |
| FeRDW | Genotype | 207 | 1997.39 | 9.65 | 30.5 | <.0001 | 96.8 |
|  | Rep | 1 | 1.14 | 1.14 | 3.6 | 0.0586 |  |
|  | Error | 207 | 65.43 | 0.32 |  |  |  |
| FeSWC | Genotype | 207 | 4917.11 | 23.75 | 1.3 | 0.0449 | 55.8 |
|  | Rep | 1 | 11.76 | 11.76 | 0.6 | 0.4292 |  |
|  | Error | 207 | 3881.85 | 18.75 |  |  |  |
| SFe | Genotype | 207 | 6811.50 | 32.91 | 10.4 | <.0001 | 90.8 |
|  | Rep | 1 | 39.07 | 39.07 | 12.4 | 0.0005 |  |
|  | Error | 207 | 653.33 | 3.16 |  |  |  |
| Fe/CKSH | Genotype | 207 | 6.65 | 0.03 | 6.4 | <.0001 | 86.4 |
|  | Rep | 1 | 0.01 | 0.01 | 1.6 | 0.205 |  |
|  | Error | 206 | 1.04 | 0.01 |  |  |  |
| Fe/CKRL | Genotype | 207 | 27.85 | 0.13 | 3.2 | <.0001 | 76.1 |
|  | Rep | 1 | 0.00 | 0.00 | 0.1 | 0.751 |  |
|  | Error | 206 | 8.75 | 0.04 |  |  |  |
| Fe/CKSFW | Genotype | 207 | 23.71 | 0.11 | 8.0 | <.0001 | 88.9 |
|  | Rep | 1 | 0.01 | 0.01 | 0.5 | 0.4728 |  |
|  | Error | 207 | 2.95 | 0.01 |  |  |  |
| Fe/CKSDW | Genotype | 207 | 17.66 | 0.09 | 6.3 | <.0001 | 86.2 |
|  | Rep | 1 | 0.02 | 0.02 | 1.2 | 0.2699 |  |
|  | Error | 207 | 2.81 | 0.01 |  |  |  |
| Fe/CKRDW | Genotype | 207 | 74.62 | 0.36 | 6.9 | <.0001 | 86.9 |
|  | Rep | 1 | 0.42 | 0.42 | 8.0 | 0.0052 |  |
|  | Error | 207 | 10.78 | 0.05 |  |  |  |
| Fe/CKSWC | Genotype | 207 | 0.75 | 0.00 | 1.4 | 0.0116 | 57.7 |
|  | Rep | 1 | 0.00 | 0.00 | 0.9 | 0.3391 |  |
|  | Error | 207 | 0.55 | 0.00 |  |  |  |

ANOVA results of all measured traits under control and Fe toxicity conditions for 211 *indica* accessions

| Trait | Source of variation | *df* | *SS* | *MS* | *F* | *P-* value | *R*^2^ (%) |
| --- | --- | --- | --- | --- | --- | --- | --- |
| SH | Genotype | 207 | 15161.93 | 73.25 | 9.80 | <.0001 | 92.0 |
|  | Environment | 1 | 2619.40 | 2619.40 | 350.37 | <.0001 |  |
|  | Error | 207 | 1547.57 | 7.48 |  |  |  |
| RL | Genotype | 207 | 788.73 | 3.81 | 1.62 | 0.0003 | 74.5 |
|  | Environment | 1 | 634.96 | 634.96 | 270.04 | <.0001 |  |
|  | Error | 207 | 486.73 | 2.35 |  |  |  |
| SFW | Genotype | 207 | 3070727.75 | 14834.43 | 9.65 | <.0001 | 92.4 |
|  | Environment | 1 | 773300.29 | 773300.29 | 503.19 | <.0001 |  |
|  | Error | 207 | 318113.75 | 1536.78 |  |  |  |
| SDW | Genotype | 207 | 57984.64 | 280.12 | 16.01 | <.0001 | 94.7 |
|  | Environment | 1 | 6712.12 | 6712.12 | 383.70 | <.0001 |  |
|  | Error | 207 | 3621.12 | 17.49 |  |  |  |
| RDW | Genotype | 207 | 1199.56 | 5.79 | 3.86 | <.0001 | 84.3 |
|  | Environment | 1 | 468.67 | 468.67 | 312.50 | <.0001 |  |
|  | Error | 207 | 310.44 | 1.50 |  |  |  |
| SWC | Genotype | 207 | 708.91 | 3.42 | 1.39 | 0.0086 | 68.8 |
|  | Environment | 1 | 413.94 | 413.94 | 168.51 | <.0001 |  |
|  | Error | 207 | 508.50 | 2.46 |  |  |  |
