# Supplementary material for: QTL mapping and candidate gene analysis of ferrous iron and zinc toxicity tolerance at seedling stage in rice by genome-wide association study
Source: BMC Genomics. 2017 Oct 27;18:828. doi: 10.1186/s12864-017-4221-5 (PMC5658907; doi:10.1186/s12864-017-4221-5)
Supplement: Supplementary file 3 — ANOVA results for the measured traits under zinc toxicity for 211 indica accessions. (DOCX 26 kb) [file 12864_2017_4221_MOESM3_ESM.docx]

**Additional file 4**

ANOVA results for the measured traits under zinc toxicity for 211 *indica* accessions

| Trait | Source of variation | df | SS | MS | F | P- value | R^2^ (%) |
| --- | --- | --- | --- | --- | --- | --- | --- |
| CKSH | Genotype | 206 | 35358.97 | 171.65 | 19.1 | <.0001 | 94.9 |
|  | Rep | 1 | 49.59 | 49.59 | 5.5 | 0.0198 |  |
|  | Error | 205 | 1843.68 | 8.99 |  |  |  |
| CKRL | Genotype | 206 | 646.19 | 3.14 | 2.9 | <.0001 | 73.5 |
|  | Rep | 1 | 8.07 | 8.07 | 7.3 | 0.0073 |  |
|  | Error | 205 | 225.41 | 1.10 |  |  |  |
| CKSFW | Genotype | 206 | 8985011.89 | 43616.56 | 5.9 | <.0001 | 85.6 |
|  | Rep | 1 | 3895.33 | 3895.33 | 0.5 | 0.4678 |  |
|  | Error | 204 | 1502054.80 | 7363.01 |  |  |  |
| CKSDW | Genotype | 206 | 154667.20 | 750.81 | 6.6 | <.0001 | 86.9 |
|  | Rep | 1 | 13.03 | 13.03 | 0.1 | 0.7355 |  |
|  | Error | 205 | 23339.82 | 113.85 |  |  |  |
| CKRDW | Genotype | 206 | 2408.09 | 11.69 | 4.0 | <.0001 | 79.5 |
|  | Rep | 1 | 15.03 | 15.03 | 5.1 | 0.0253 |  |
|  | Error | 205 | 606.49 | 2.96 |  |  |  |
| CKSWC | Genotype | 206 | 649.80 | 3.15 | 1.4 | 0.0048 | 59.2 |
|  | Rep | 1 | 0.58 | 0.58 | 0.3 | 0.6068 |  |
|  | Error | 204 | 447.36 | 2.19 |  |  |  |
| ZnSH | Genotype | 206 | 19016.48 | 92.31 | 14.3 | <.0001 | 93.5 |
|  | Rep | 1 | 0.00 | 0.00 | 0.0 | 0.9963 |  |
|  | Error | 206 | 1326.12 | 6.44 |  |  |  |
| ZnRL | Genotype | 206 | 1368.73 | 6.64 | 6.4 | <.0001 | 86.4 |
|  | Rep | 1 | 2.22 | 2.22 | 2.2 | 0.144 |  |
|  | Error | 206 | 212.52 | 1.03 |  |  |  |
| ZnSFW | Genotype | 206 | 2644873.46 | 12839.19 | 6.1 | <.0001 | 85.0 |
|  | Rep | 1 | 35209.68 | 35209.68 | 16.8 | <.0001 |  |
|  | Error | 206 | 431823.69 | 2096.23 |  |  |  |
| ZnSDW | Genotype | 206 | 57272.53 | 278.02 | 5.4 | <.0001 | 82.3 |
|  | Rep | 1 | 1628.59 | 1628.59 | 31.5 | <.0001 |  |
|  | Error | 206 | 10647.13 | 51.69 |  |  |  |
| ZnRDW | Genotype | 206 | 1661.15 | 8.06 | 4.2 | <.0001 | 80.0 |
|  | Rep | 1 | 17.77 | 17.77 | 9.2 | 0.0027 |  |
|  | Error | 206 | 397.28 | 1.93 |  |  |  |
| ZnSWC | Genotype | 206 | 1162.19 | 5.64 | 2.4 | <.0001 | 70.6 |
|  | Rep | 1 | 3.87 | 3.87 | 1.7 | 0.1984 |  |
|  | Error | 206 | 479.39 | 2.33 |  |  |  |
| SZn | Genotype | 206 | 385.74 | 1.87 | 1.8 | <.0001 | 64.8 |
|  | Rep | 1 | 0.53 | 0.53 | 0.5 | 0.4706 |  |
|  | Error | 206 | 209.19 | 1.02 |  |  |  |
| Zn/CKSH | Genotype | 206 | 2.92 | 0.01 | 2.3 | <.0001 | 69.6 |
|  | Rep | 1 | 0.03 | 0.03 | 5.4 | 0.0208 |  |
|  | Error | 205 | 1.24 | 0.01 |  |  |  |
| Zn/CKRL | Genotype | 206 | 11.76 | 0.06 | 2.4 | <.0001 | 69.5 |
|  | Rep | 1 | 0.18 | 0.18 | 7.2 | 0.0078 |  |
|  | Error | 205 | 4.99 | 0.02 |  |  |  |
| Zn/CKSFW | Genotype | 206 | 13.31 | 0.06 | 2.3 | <.0001 | 69.7 |
|  | Rep | 1 | 0.06 | 0.06 | 2.0 | 0.1625 |  |
|  | Error | 204 | 5.72 | 0.03 |  |  |  |
| Zn/CKSDW | Genotype | 206 | 23.55 | 0.11 | 1.4 | 0.0072 | 58.6 |
|  | Rep | 1 | 0.09 | 0.09 | 1.1 | 0.288 |  |
|  | Error | 204 | 16.54 | 0.08 |  |  |  |
| Zn/CKRDW | Genotype | 206 | 24.38 | 0.12 | 2.2 | <.0001 | 68.7 |
|  | Rep | 1 | 0.00 | 0.00 | 0.0 | 0.9636 |  |
|  | Error | 204 | 11.10 | 0.05 |  |  |  |
| Zn/CKSWC | Genotype | 206 | 0.20 | 0.00 | 1.6 | 0.0002 | 62.1 |
|  | Rep | 1 | 0.00 | 0.00 | 2.1 | 0.1499 |  |
|  | Error | 204 | 0.12 | 0.00 |  |  |  |

ANOVA results for all the measured traits under both control and zinc toxicity conditions for 211 *indica* accessions

| Trait | Source of variation | *df* | *SS* | *MS* | *F* | *P-* value | *R*^2^ (%) |
| --- | --- | --- | --- | --- | --- | --- | --- |
| SH | Genotype | 206 | 25060.89 | 121.65 | 11.33 | <.0001 | 94.6 |
|  | Environment | 1 | 13454.85 | 13454.85 | 1253.01 | <.0001 |  |
|  | Error | 206 | 2212.03 | 10.74 |  |  |  |
| RL | Genotype | 206 | 712.34 | 3.46 | 2.40 | <.0001 | 75.1 |
|  | Environment | 1 | 180.22 | 180.22 | 125.15 | <.0001 |  |
|  | Error | 206 | 296.65 | 1.44 |  |  |  |
| SFW | Genotype | 206 | 4469143.69 | 21694.87 | 3.35 | <.0001 | 84.8 |
|  | Environment | 1 | 2986383.40 | 2986383.40 | 461.59 | <.0001 |  |
|  | Error | 206 | 1332763.59 | 6469.73 |  |  |  |
| SDW | Genotype | 206 | 85140.48 | 413.30 | 5.19 | <.0001 | 87.1 |
|  | Environment | 1 | 26045.50 | 26045.50 | 327.16 | <.0001 |  |
|  | Error | 206 | 16399.86 | 79.61 |  |  |  |
| RDW | Genotype | 206 | 1513.91 | 7.35 | 4.59 | <.0001 | 86.5 |
|  | Environment | 1 | 599.92 | 599.92 | 374.51 | <.0001 |  |
|  | Error | 206 | 329.99 | 1.60 |  |  |  |
| SWC | Genotype | 206 | 497.10 | 2.41 | 1.28 | 0.0367 | 78.4 |
|  | Environment | 1 | 907.89 | 907.89 | 483.14 | <.0001 |  |
|  | Error | 206 | 387.10 | 1.88 |  |  |  |
