# Supplementary material for: QTL mapping and candidate gene analysis of ferrous iron and zinc toxicity tolerance at seedling stage in rice by genome-wide association study
Source: BMC Genomics. 2017 Oct 27;18:828. doi: 10.1186/s12864-017-4221-5 (PMC5658907; doi:10.1186/s12864-017-4221-5)
Supplement: Supplementary file 4 — Correlations between all measured traits under control (upper triangular) and stress (lower triangular) conditions in Fe (a) and Zn (b) experiments. (DOCX 275 kb) [file 12864_2017_4221_MOESM4_ESM.docx]

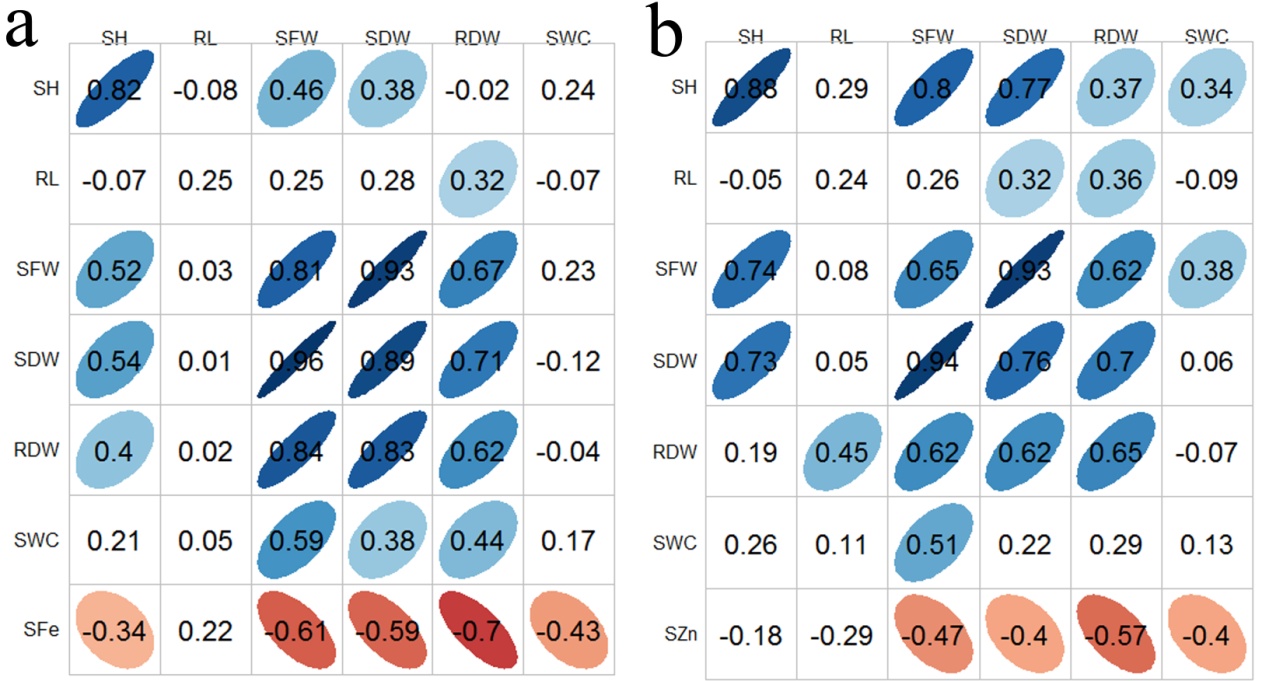


**Additional file 2:** Correlations between all measured traits under control (upper triangular) and stress (lower triangular) conditions in Fe (a) and Zn (b) experiments, respectively. The values on the principal diagonal were correlation coefficients of the same trait between control and stress conditions. Ellipses obliquing to right and left indicated positive and negative correlations, respectively. The values without glyphs indicated insignificant at 0.05. SH, Shoot height; RL, Root length; SFW, Shoot fresh weight; SDW, Shoot dry weight; RDW, Root dry weight; SWC, Shoot water content; SFe, Fe concentration in shoot; SZn, Zn concentration in shoot.
